# Biological Applications of Functionalized Cerium Oxide Nanoparticles

**DOI:** 10.7150/ijms.134122

**Published:** 2026-05-29

**Authors:** Siyu Chen, Yuqi Gong, Fagang Jiang, Xinghua Wang

**Affiliations:** Department of Ophthalmology, Union Hospital, Tongji Medical College, Huazhong University of Science and Technology, Wuhan, Hubei Province, China.

**Keywords:** cerium oxide nanoparticles, biomedical applications, oxidative stress, toxicity

## Abstract

The rapid expansion and advancement of nanotechnology have presented potential applications in the diagnosis and treatment of various diseases. In recent years, cerium oxide nanoparticles (CeO_2_ NPs) have been extensively studied and applied in biological systems due to their unique physical and chemical properties, such as high antioxidant activity, enzyme-mimicking activity and good biocompatibility. This review summarizes the latest research progress of functionalized CeO_2_ NPs in anti-cancer activity, anti-infection, anti-inflammation, antioxidant activity, wound healing, neuroprotection and imaging applications. It explores their mechanisms of action and potential clinical application prospects, as well as the concerns about toxicity and safety.

## 1. Introduction

### 1.1. Research Background: The Rise of CeO_2_ NPs

**Figure 1:**
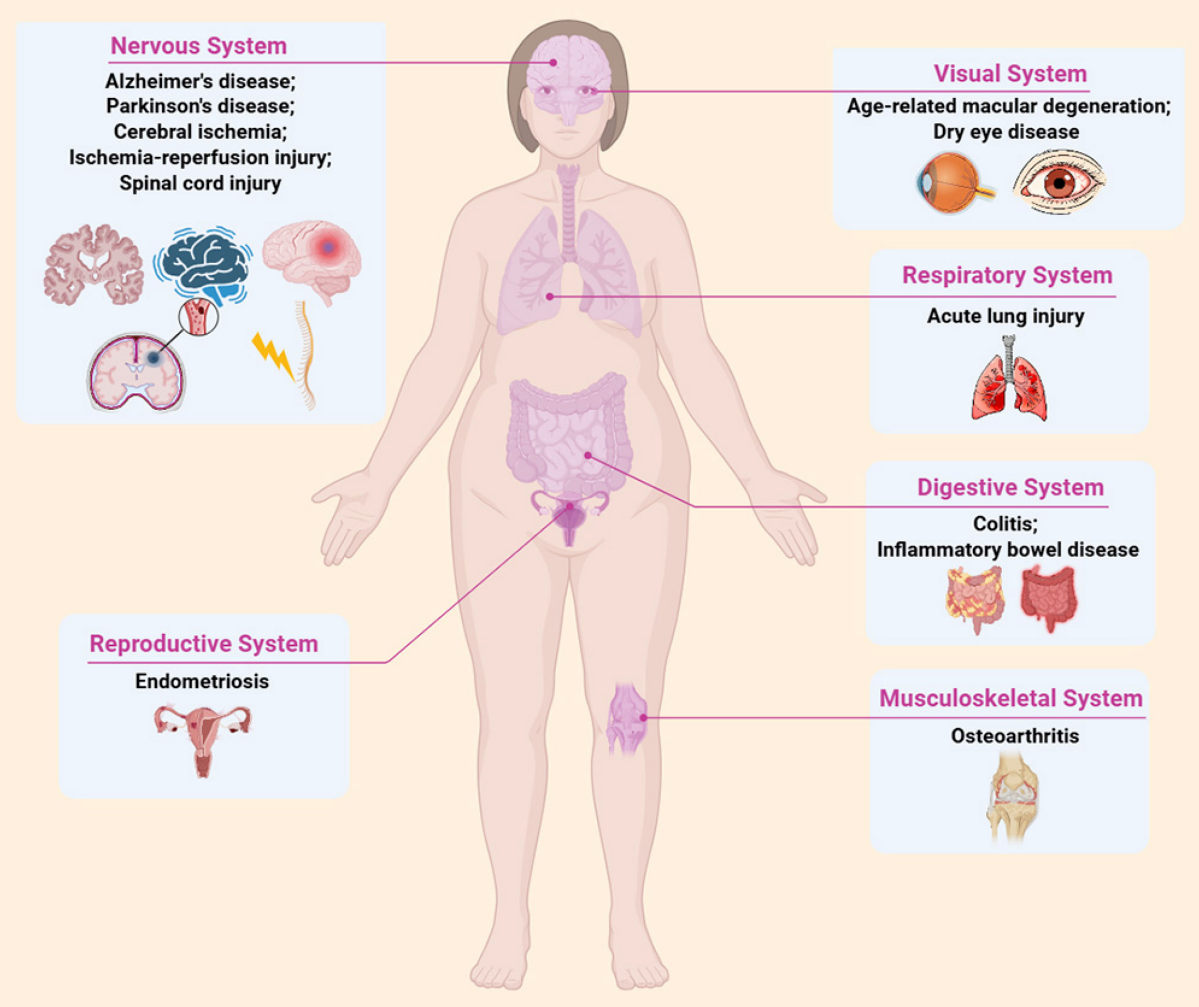
Applications of functionalized cerium oxide nanoparticles in disease treatment. Created with BioRender.com.

The rapid growth and development of nanotechnology have provided potential applications for the diagnosis and treatment of various diseases 1 (**Figure 1**). Among the numerous nanomaterials under intensive research, gold nanoparticles (Au NPs), silica nanoparticles (SiO_2_ NPs), iron oxide nanoparticles (IONPs), and polymer-based nanocarriers are the most commonly studied types, each with distinct advantages and inherent limitations. Specifically, Au NPs possess excellent optical properties and high biocompatibility, making them outstanding candidates for bioimaging and photothermal therapy, but their high synthesis cost and poor biodegradability restrict their large-scale clinical application 2. SiO_2_ NPs have a stable spherical structure, large specific surface area, and good biocompatibility, which make them ideal drug delivery carriers, yet their poor biodegradability may lead to long-term accumulation in the body and potential toxicity 3. IONPs exhibit unique superparamagnetic properties, enabling their application in magnetic resonance imaging (MRI)^4^, but their antioxidant capacity is relatively weak, limiting their efficacy in treating oxidative stress-related diseases. Polymer-based nanocarriers, such as liposomes and polymeric micelles, have flexible structural design and good drug loading capacity, but they are prone to degradation and instability in the complex *in vivo* environment, affecting their delivery efficiency 5.

**Figure 2:**
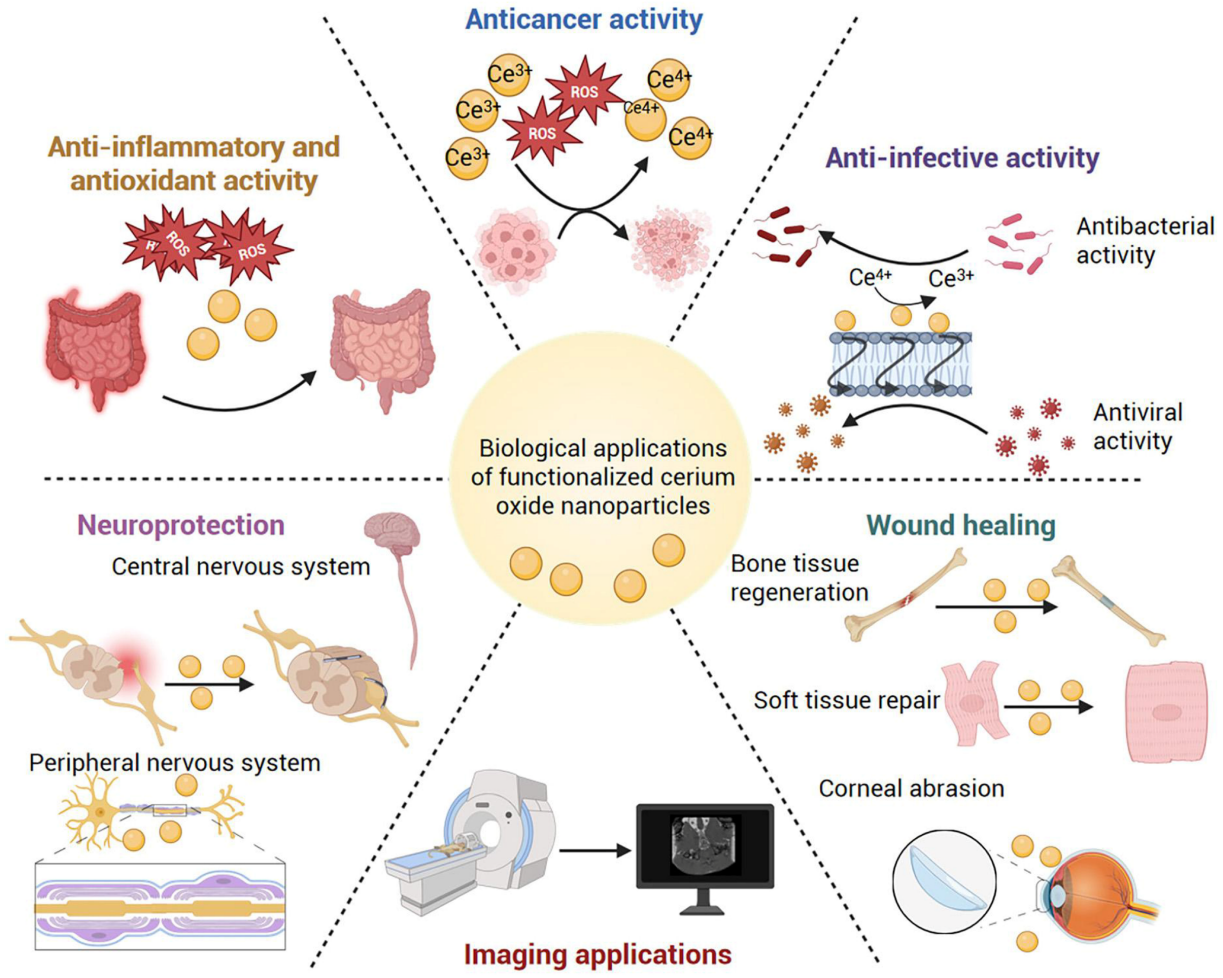
Biological applications of functionalized cerium oxide nanoparticles. Created with BioRender.com.

Unlike these commonly used nanomaterials, CeO_2_ NPs have attracted significant attention over the past decade due to their unique intrinsic biological effects, including high antioxidant activity 6, enzyme-mimicking activity 7,8, and biocompatibility 9. In recent years, increasing evidence suggests that CeO_2_ NPs have broad application prospects in the biomedical field 10 (**Figure 2**), and their unique properties have shown good potential in antioxidant, antitumor, anti-infection, and neuroprotective aspects.

The unique biological effects of CeO_2_ NPs are closely related to their three-core structural and chemical characteristics: reversible Ce^3+^/Ce^4+^ redox cycle, oxygen vacancies, and adjustable surface chemical states. The reversible conversion between Ce^3+^ and Ce^4+^ on the surface of CeO_2_ NPs allows them to continuously accept and donate electrons, thereby efficiently scavenging various reactive oxygen species (ROS) (such as superoxide anion, hydroxyl radical, and hydrogen peroxide) in biological systems and maintaining the redox balance of cells 11. Oxygen vacancies, as important surface defects, further provide active sites that synergistically enhance the enzyme-mimicking activity (e.g., superoxide dismutase (SOD) and catalase (CAT)) and overall antioxidant capacity of CeO_2_ NPs 12.

Notably, the modulation of these core structural and chemical characteristics is closely associated with the synthesis methods and functionalization strategies of CeO_2_ NPs, which can provide a more solid mechanistic basis for their unique biological effects. Specifically, functionalization strategies have been proven to significantly regulate the Ce^3+^/Ce^4+^ ratio and the catalytic performance of CeO_2_ NPs. Compositional modulation, such as transition metal doping (e.g., Fe, Cu), can alter the electronic cloud distribution around Ce atoms, promote the conversion of Ce^4+^ to Ce^3+^, and increase the Ce^3+^ content on the material surface. Meanwhile, structural defect engineering, such as the introduction of oxygen vacancies, can effectively increase oxygen vacancy density, which in turn synergistically enhances the redox activity of the Ce^3+^/Ce^4+^ cycle and optimizes the ROS scavenging capacity of CeO_2_ NPs 13.

### 1.2. The Necessity of Functional Modification: Addressing Issues of Aqueous Dispersibility, Targeting Capability, Biocompatibility, and Toxicity

CeO_2_ NPs exhibit a significant tendency to agglomerate, resulting in poor stability and dispersion in aqueous media 14. Agglomerated particles may impair their distribution and metabolism within the body, increasing potential toxicity risks 15. Functionalization modifications can improve their dispersion and stability 16. For example, surface functionalization with polyvinylpyrrolidone (PVP) can effectively improve the dispersion stability of CeO_2_ nanoparticles and prevent their aggregation 17.

**Figure 3:**
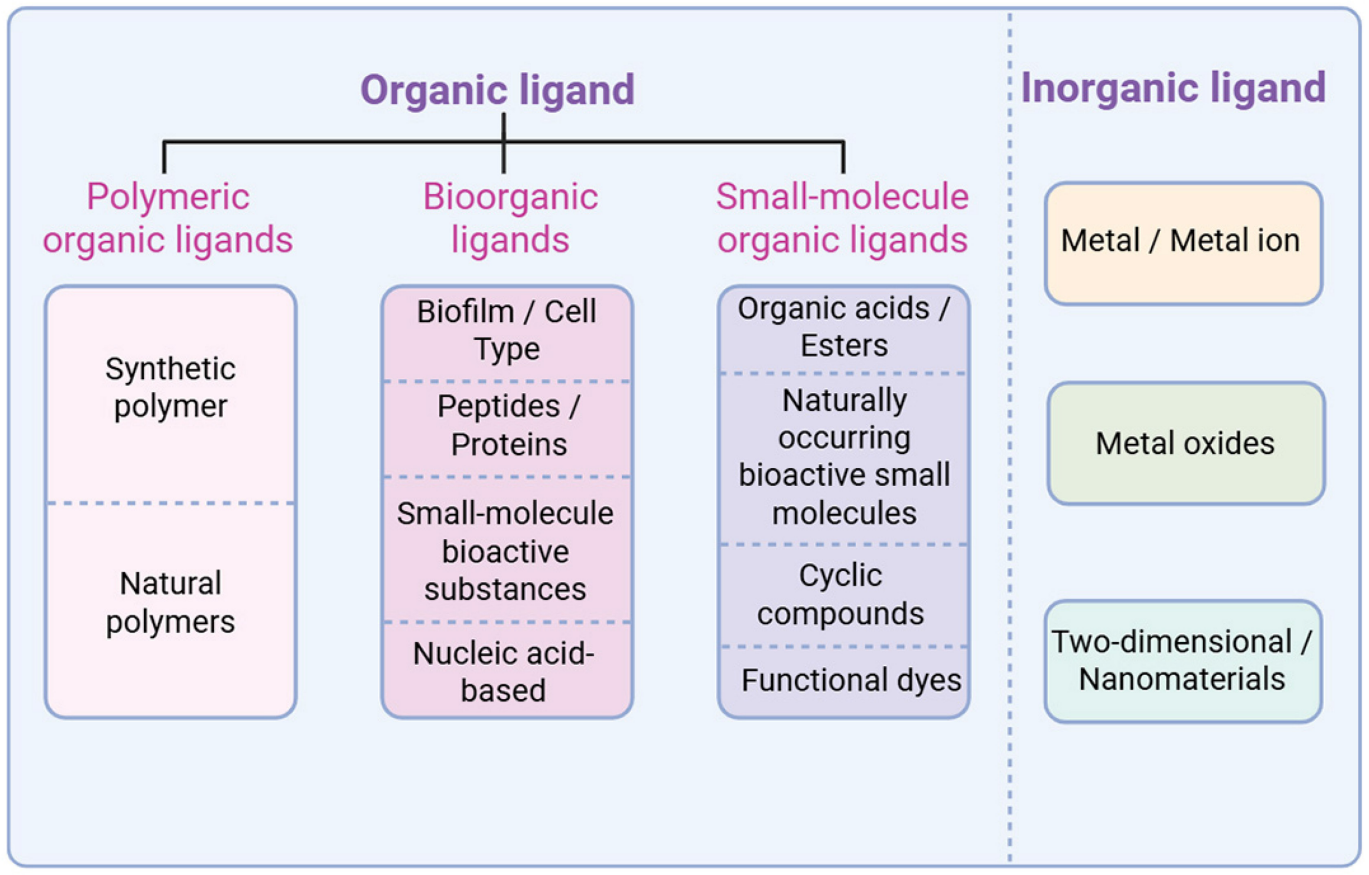
Functionalized cerium oxide nanoparticle-modified ligands. Created with BioRender.com.

In the biomedical field, targeted functionalization of CeO_2_ NPs is essential to enable more precise delivery to diseased sites, enhance therapeutic efficacy, and minimize damage to healthy tissues. For instance, specific targeting molecules, including antibodies or ligands (**Figure 3**), can be anchored on the CeO_2_ NPs surface. This allows the CeO_2_ NPs to recognize and bind specifically to receptors on diseased cells or tissues, thereby achieving targeted delivery. Although related research remains relatively scarce, targeted modification represents one of the critical steps for realizing precision therapy with CeO_2_ NPs in biomedical applications.

Uncoated CeO_2_ NPs exhibit limited biocompatibility 18, as their biopersistence and tendency to accumulate in mononuclear phagocytic system organs can lead to increased toxicity over time, including oxidative stress, inflammation, and tissue damage 19. Surface functionalization can improve the properties of CeO_2_ NPs, reducing adverse reactions with biological systems. For instance, by modifying CeO_2_ NPs with lipoic acid (LA) and polyethylene glycol (PEG), it is possible to prepare an LA-PEG-CeO_2_ nanocomposite system that exhibits good biocompatibility, low toxicity, and high free radical scavenging capacity 20.

High concentrations of CeO_2_ NPs have been shown to induce cytotoxicity and genotoxicity *in vitro*, raising safety concerns for potential *in vivo* applications 21. Functionalization modifications can reduce toxicity by altering CeO_2_ NPs' surface charge, particle size, and surface chemical composition. For example, *in situ* growth of CeO_2_ on negatively charged montmorillonite sheets optimizes surface properties and colloidal stability, effectively reducing systemic absorption and potential nanotoxicity for safer biomedical use 22.

### 1.3. Functional Modification Methods

The methods for functionally modifying CeO_2_ NPs using nanotechnology include surface chemical modification (including organic molecule modification, coupling agent modification, surfactant modification, as well as novel bioorganic ligand modification represented by elastin-based protein functionalization), surface physical modification (including polymer coating, metal nanoparticle coating, etc.), and *in situ* synthesis. **Table 1** and **Table 2** respectively present typical functionalization strategies for CeO_2_ NPs and their synthesis methods in different application scenarios. Functional modification is capable of improving their dispersion and stability, enhancing biocompatibility and targeting ability, boosting mechanical properties, increasing application efficiency, or endowing CeO_2_ NPs with novel physical and chemical characteristics. Figure 3 shows the ligands of functionalized CeO_2_ NPs.

### 1.4. Heterostructure Engineering of CeO_2_-Based Nanozymes

Heterostructure engineering is a pivotal design strategy to elevate the catalytic and enzyme-mimetic performance of CeO_2_ nanozymes 23. By integrating CeO_2_ with transition metal oxides, rare-earth solids or noble metals to form well-defined interfaces, this strategy realizes multi-dimensional regulation of lattice strain, oxygen vacancy, component composition and interfacial electronic synergy, and optimizes the Ce^3+^/Ce^4+^ redox cycle at the atomic level to break the activity limit of pristine CeO_2_^24^.

Lattice strain induced by heteroepitaxy can effectively boost oxygen vacancy generation 25. This mechanism, combined with the strategy of component complementation which broadens enzyme-mimetic adaptability 26, has been successfully applied to design high-performance ceria-based heterostructures for treating ROS-related diseases. For instance, epitaxially strained CeO_2_/Mn_3_O_4_ nanocrystals leverage enhanced oxygen vacancies and redox cycles to achieve superior radioprotection 27. Similarly, CeO_2_/MoS_2_ heterostructures optimize these properties to enhance ROS scavenging capacity, anti-inflammatory effects, antibacterial action, and wound healing 28. In a different direction, CeO_2_/Au@Pt nanospheres utilize dual enzyme-mimetic activities (peroxidase-like (POD-like) and CAT-like) to generate cytotoxic hydroxyl radicals and alleviate tumor hypoxia, enabling photothermal-catalytic synergistic therapy of tumors 29.

Compared with conventional modification, heterostructure engineering provides systematic lattice-electronic-compositional synergy, greatly expanding the biomedical applications of CeO_2_-based nanomedicines 23. Through precise interface regulation and electronic structure optimization, these representative heterostructure designs effectively tailor the catalytic behavior of CeO_2_ nanozymes. As summarized in **Table 3**, such structural engineering endows CeO_2_ nanozymes with enhanced performance and extended functional potential in biomedicine.

As summarized in **Table 4**, different functionalization strategies exert distinct regulatory effects on the three major enzyme-mimetic activities of CeO_2_ NPs. Element doping (e.g., Cu, Pt) primarily enhances oxygen vacancy density and Ce^3+^/Ce^4+^ redox cycling, thereby boosting SOD/CAT-like activities (for ROS scavenging) or POD-like activity (for pro-oxidant therapy). Surface modification (e.g., with polymers, peptides, or polysaccharides) mainly improves colloidal stability, targeting, and biocompatibility while preserving or moderately enhancing intrinsic ROS-scavenging activities. Surface facet engineering further reveals that exposed crystal facets dictate catalytic preference, with {110} facets favoring SOD-like activity and {100} facets favoring POD-like activity 30. Collectively, these strategies allow tailored design of CeO_2_ nanozymes for either antioxidant therapy (SOD/CAT-dominant) or pro-oxidant therapy (POD-dominant), depending on the pathological context.

## 2. Biomedical Applications of Functional CeO_2_ NPs

### 2.1. Anticancer Activity

#### 2.1.1. Inhibition of Tumor Growth

CeO_2_ NPs have the capability to suppress tumor growth by triggering apoptosis in tumor cells 31. Their core mechanisms of action include modulating the tumor microenvironment (TME) (e.g., scavenging excess ROS in normal tissues or generating ROS in acidic TME, enabling targeted delivery of therapeutics through surface functionalization to enhance selective killing of tumor cells and reduce damage to normal cells.

Engineering TME-responsive catalytic systems is critical for improving the selectivity and efficacy of nanocatalytic cancer therapy. A pH-responsive PN-CeO_2_-PSS system was prepared by electrostatically conjugating porous CeO_2_ nanorods with PSS, exerting catalytic activity only in acidic TME 32. Its nanoreactor structure traps oxygen and substrates, promotes charge transfer, and efficiently generates ·O_2_^-^, which induces oxidative stress (membrane damage, elevated malondialdehyde (MDA)), activates caspase-3-mediated tumor cell apoptosis and inhibits tumor growth. 70 kDa PSS modification was optimal: PN-CeO_2_-PSS with a hydrodynamic diameter of 306.5 nm balanced substrate capture and charge transfer efficiency, showing the highest oxidative and antitumor activity. It inhibited HepG2 tumors at 96.1% (vs. 51.1% for unmodified PN-CeO_2_), with no toxicity to normal cells/mouse organs, excellent biocompatibility and metabolic safety. This pH-responsive polymer-CeO_2_ hybrid provides a highly selective, effective and low-toxic nanocatalytic platform for tumor chemodynamic therapy (CDT), offering important insights for nanomedicine clinical translation.

For breast cancer nanotherapy, developing a smart-responsive, synergistic and low-toxic nanomedicine system is key to overcoming the drawbacks of traditional CeO_2_-based nanotherapeutics (weak POD-mimetic activity, poor monotherapy efficacy) and chemotherapeutics (severe toxic side effects). A study fabricated the smart-responsive CCCs nanosystem via hydrothermally doping Cu^2+^ into CeO_2_ lattice, electrostatically loading DOX on Cu-CeO_2_ surface and ultrasonicating MDA-MB-231 cell membrane coating 33. Cu-CeO_2_ NPs show enhanced POD-mimetic activity in acidic TME, generating toxic OH via Fenton-like reactions to induce tumor cell apoptosis. CCCs realize precise tumor targeting and pH-responsive DOX release, achieving synergistic tumor suppression with CDT; cell membrane coating endows it with targeting ability and biocompatibility. *In vitro*, 200 μg/mL CCCs kill 95.8% of MDA-MB-231 cells with low cytotoxicity to C2C12 cells. *In vivo*, it inhibits 98.5% of tumor growth without obvious organ damage or abnormal blood parameters, realizing highly effective and low-toxic breast cancer treatment. Moreover, the system exerts antioxidant activity under normal physiological conditions, scavenging excess ROS to alleviate DOX-induced normal tissue damage and ensure treatment safety and sustainability.

In head and neck cancer nanotherapy, surface modification to regulate nanoparticle bioactivity and targeting is key for optimizing efficacy, with modifier molecular weight drawing much attention. Tarakci *et al*. 34 synthesized two Dex-CeO_2_ NPs (SD1: 9-11 kDa dextran; SD2: 6 kDa dextran). SD2 markedly elevated intracellular ROS at high concentrations, inducing apoptosis. Cytotoxicity assays on A253, FaDu and SCC-25 cells showed SD2 exerted high cytotoxicity at 500/1000 µg/mL, significantly reducing viability. Gene expression analysis revealed that SD2 exerted anticancer effects by upregulating pro-apoptotic genes (TP53, CASP3, BAX), contributing to tumor cell apoptosis.

#### 2.1.2. Enhancing Radiotherapy Effects

When used in combination with radiotherapy, CeO_2_ NPs can increase the sensitivity of tumor cells to radiotherapy while reducing damage to normal cells 35.

A core challenge in improving radiotherapy efficacy and safety is balancing precise tumor radiosensitization with normal tissue protection, where cell-type-selective redox-regulating nanotherapeutics are key. Zamyatina *et al*. 36 developed pyrroloquinoline quinone-modified CeO_2_ NPs (CeO_2_@PQQ NPs). These NPs enhance cancer cell (EMT6/P) radiosensitivity to X-rays by inducing oxidative stress: post 5 Gy irradiation, glutathione (GSH) levels (key antioxidant) decreased by 32%, impairing ROS scavenging and accumulating oxidative damage. They also disrupted mitochondrial function, reducing mitochondrial membrane potential (MMP) by 29% to trigger pro-apoptotic pathways. CMN assay showed a 2.5-fold increase in micronuclei (chromosomal damage marker), and clonogenic assay revealed a 2-fold reduced survival fraction, exacerbating DNA damage and inhibiting repair. In contrast, for normal L929 cells, CeO_2_@PQQ NPs post-radiotherapy maintained viability, elevated GSH and MMP, reduced micronuclei, and enhanced survival, exerting radioprotection. With cell-type-specific antioxidant/pro-oxidant properties, CeO_2_@PQQ NPs lay a theoretical foundation for novel radiosensitizers/antioxidants.

Radiotherapy resistance induced by tumor hypoxia, the lack of precision guidance during treatment, and inadequate control of radiotherapy toxicity are key issues limiting the improvement of radiotherapy efficacy for solid tumors. The construction of an integrated nanoplatform with TME responsiveness, hypoxia alleviation, efficacy enhancement, and imaging guidance functions is of great significance for addressing the aforementioned problems. Pi *et al*. 37 developed core-shell CeO_2_-MnO_2_ nanoparticles, a novel radiosensitizer with excellent biocompatibility and clinical translation potential, enhancing radiotherapy efficacy while reducing toxicity. *In vitro*, MnO_2_ catalyzes O_2_ generation from TME H_2_O_2_ (alleviating hypoxia) and depletes GSH (weakening antioxidant defenses), while CeO_2_ boosts X-ray absorption and synergistically generates ROS to increase radiosensitivity. Mn^2+^ from MnO_2_ degradation enhances T1-weighted MRI signal (peak at 4 h post-injection) for tumor localization and guidance. *In vivo* 21-day experiments showed CeO_2_-MnO_2_ + X-rays significantly reduced tumor volume, with no toxicity to normal MIHA cells or mouse major organs, and normal blood/biochemical parameters. Integrating TME responsiveness, hypoxia alleviation, and MRI guidance, CeO_2_-MnO_2_ provides a new strategy for effective, low-toxicity solid tumor radiotherapy.

#### 2.1.3. Tumor Targeting Ability

The tumor targeting capacity of CeO_2_ NPs can be enhanced by modifying their surfaces, such as through conjugation with specific peptides. These modifiers are capable of specifically binding to certain receptors or molecules on tumor cell surfaces, which directs CeO_2_ NPs to reach tumor cells and promotes their accumulation at the tumor site.

The insufficient tumor targeting of nanophototherapeutic agents is a key bottleneck leading to low precision in photothermal therapy for breast cancer and a high risk of thermal damage to normal tissues. Constructing an integrated nanozyme with "active targeting-photothermal conversion-imaging guidance" functions has become a core direction for improving treatment accuracy and safety. Researchers successfully developed a targeted nanozyme (HCeO_2_@ICG-RGD) by loading indocyanine green (ICG) onto mesoporous cerium oxide nanocatalysts (HCeO_2_) and modifying the surface of HCeO_2_@ICG with arginine-glycine-aspartic acid (RGD) peptides, enabling specific recognition and efficient photothermal therapy for breast cancer 38. This peptide exhibits specific recognition and binding to αvβ3 integrins on tumor cell surfaces, thereby enabling targeted identification of tumor cells and enhancing their targeting efficiency. The targeting property of HCeO_2_@ICG-RGD in tumor tissue was studied by near-infrared-II (NIR II) fluorescence imaging, and results showed that HCeO_2_@ICG-RGD has good targeting property. The results from confocal laser scanning microscopy (CLSM) also showed that the amount of RGD modified HCeO_2_@ICG-RGD nanozymes absorbed by 4T1 breast cancer cells was greatly increased compared with HCeO_2_@ICG.It proved that the RGD peptide could enhance the targeting ability of the nanozymes for tumor cells. In photothermal therapy, CeO_2_ NPs can absorb light energy of a specific wavelength and convert it into heat. When they accumulate in the tumor site, they can increase the local temperature of the tumor through external light source irradiation, thereby killing tumor cells. Its targeting ability for tumor can improve the accuracy and effectiveness of photothermal therapy and reduce the thermal damage for the surrounding normal tissues, and it has certain application value in cancer treatment, and also provides new ideas and methods for cancer treatment.

#### 2.1.4. Reversal of Tumor Drug Resistance

Abnormally elevated intracellular Ca^2+^ levels are intimately associated with tumor drug resistance. High Ca^2+^ concentrations can activate drug efflux proteins (such as P-glycoprotein (P-gp)), prompting the efflux of chemotherapeutic agents from tumor cells and consequently diminishing drug efficacy.

A study conducted by Tian *et al*. 39 engineered phytic acid (PA)-modified cerium dioxide nanoparticles (CeO_2_@PA). Leveraging PA's robust coordination capacity with Ca^2+^, the nanoparticles enable effective modulation of intracellular Ca^2+^ levels. This intervention reduces intracellular Ca^2+^ concentrations, inhibits P-gp expression, and markedly enhances drug accumulation in drug-resistant tumor cells, thereby achieving efficient reversal of tumor drug resistance. This study indicates that CeO_2_@PA is expected to be a new type of Ca^2+^ inhibitor for reversing tumor drug resistance, providing new strategies and methods for overcoming tumor drug resistance.

### 2.2. Anti-Infection

#### 2.2.1. Antibacterial Activity

The spread of multidrug-resistant bacteria has greatly reduced the efficacy of traditional antibiotics, urgently calling for novel high-efficiency antimicrobial materials. CeO_2_ features reversible Ce^3+^/Ce^4+^ conversion, which enables in-situ ROS generation for bacterial inhibition without UV irradiation, yet pure CeO_2_ nanotiles show limited antimicrobial activity. Graphene's high conductivity and sharp-edge structure can synergistically boost the antibacterial properties of metal oxides. Accordingly, Rehman *et al*. 40 synthesized CeO_2_ nanotiles and graphene/cerium oxide (G/CeO_2_) nanocomposites via a solvothermal method to explore their synergistic antibacterial effects. The results show that the incorporation of graphene significantly enhances antimicrobial activity, with the 25% graphene-loaded G/CeO_2_-II performing optimally, achieving inhibition rates of 82.67% against P. aeruginosa and 89.48% against S. aureus. The excellent antibacterial activity of G/CeO_2_-II mainly comes from the unique nanostructures of graphene and CeO_2_ nanotiles and their synergy: graphene's sharp edges damage bacterial cell membranes and induce charge imbalance; CeO_2_'s reversible Ce^3+^/Ce^4+^ conversion generates ROS to oxidize bacterial proteins and nucleic acids; the blade-like CeO_2_ nanotiles physically pierce bacterial cell membranes. This triple synergy is believed to contribute to bacterial death. G/CeO_2_ nanocomposites show superior inhibitory effects against Gram-positive S. aureus in comparison with Gram-negative P. aeruginosa, owing to structural differences in bacterial cell membranes. Gram-positive bacteria possess a thicker peptidoglycan layer, which renders them more vulnerable to physical damage. As a promising candidate for combating drug-resistant bacteria, G/CeO_2_ nanocomposites are expected to promote technological advances in the fields of nanomedicine and anti-infection therapy.

Studies have shown that MoS_2_-CeO_2_ nanocomposites formed by combining CeO_2_ NPs with polyethylene glycol-modified molybdenum disulfide (PEG-MoS_2_) exhibit excellent photothermal antibacterial properties under 808 nm laser irradiation. CeO_2_ NPs have intrinsic antibacterial activity, derived from surface oxygen vacancies and ROS generation via reversible Ce^3+^/Ce^4+^ redox reactions; PEG-MoS_2_ converts near-infrared (NIR) light energy into heat, disrupting bacterial cell membranes through photothermal effects to exert antibacterial action. The combination yields a distinct synergistic effect, where CeO_2_ NPs' antibacterial property and PEG-MoS_2_'s photothermal antibacterial capacity reinforce each other, greatly boosting overall antimicrobial efficacy. This nanocomposite can alleviate two key issues in chronic wounds, namely infection and oxidative stress. It holds great application potential for diabetic ulcers and offers an efficient, convenient and innovative strategy for clinical chronic wound treatment 28.

#### 2.2.2. Antiviral Activity

The invasion mechanisms of enveloped viruses (such as pH-dependent fusion in endocytic pathways and dual-pathway invasion) represent key targets for antiviral material design. CeO_2_ NPs, with good biocompatibility and antiviral potential, are a research focus, but bare CeO_2_ NPs suffer from insufficient stability and limited targeting, restricting therapeutic efficacy. To address this, Dupkalová *et al*. 41 functionalized CeO_2_ NPs with essential amino acids histidine (CH) and glycine (CG), both forming stable bonds with CeO_2_ via carboxyl groups. Notably, histidine's imidazole ring exhibits pH-responsive protonation, matching the acidic endosomal microenvironment (pH 5.8-6.2) during invasion of enveloped viruses like vesicular stomatitis virus (VSV). By introducing “histidine-mediated low-pH protonation” into surface design, the team triggered nanoparticle aggregation and charge reversal in the viral fusion acidic environment, achieving an “endosome-targeting-fusion-blocking” strategy. This simple amino acid functionalization enhanced CeO_2_ NPs' therapeutic index against VSV by 1-2 orders of magnitude without increasing toxicity. The study revealed that antiviral activity strongly depends on viral entry pathways: it is highest for the endosomal pathway of VSV, intermediate for the dual pathways of bovine beta coronavirus (BCoV-1), and minimal for the non-endosomal pathway of herpes simplex virus (HSV), laying a foundation for “pathway-selective” nanovirals. Functionalization (especially histidine) significantly boosts CeO_2_ NPs' ability to capture viruses and block fusion in acidic endosomes, showing high therapeutic potential against enveloped viruses (e.g., VSV) relying on acidic endosomal conditions (pH 5.8-6.2) for membrane fusion and entry.

### 2.3. Anti-inflammatory and antioxidant activity

#### 2.3.1. Colitis and Inflammatory Bowel Disease (IBD)

ROS are key drivers in the pathological progression of colitis and IBD. Their excessive accumulation exacerbates the cycle of intestinal injury and inflammation, making them important therapeutic targets. Consequently, ROS scavengers offer a promising comprehensive strategy for effective treatment.

CeO_2_ NPs with their enzyme-mimicking activity (mimicking CAT, POD, and SOD), efficiently scavenge various ROS while exhibiting excellent biocompatibility, making them ideal candidates for inflammation treatment. To further optimize dispersion, stability, and *in vivo* targeting, Yang *et al*. 42 synthesized PEG-modified hollow CeO_2_ NPs (H-CeO_2_-PEG) via in-situ CeO_2_ growth on SiO_2_ NPs, subsequent SiO_2_ core removal, and PEG surface modification. Their study first revealed that H-CeO_2_-PEG alleviates colitis by inhibiting the MAPK signaling pathway (ERK1/2, JNK, p38, c-Jun). Possessing exceptional ROS-scavenging capacity, H-CeO_2_-PEG converts hydroxyl radicals (•OH), hydrogen peroxide (H_2_O_2_), and superoxide anions (•OOH) into water and oxygen. In a dextran sulfate sodium (DSS)-induced colitis mouse model, it significantly mitigates symptoms through multiple mechanisms: scavenging ROS, suppressing pro-inflammatory cytokines (IL-6, TNF-α, IL-1β, IL-18), and inhibiting MAPK pathway activation. Additionally, H-CeO_2_-PEG exhibits good *in vivo* biosafety, with no significant toxicity to mice's major organs at therapeutic doses, laying an important foundation for clinical applications.

Ulcerative colitis (UC) is a chronic non-specific IBD. Traditional drugs for UC are severely limited by their poor targeting, low bioavailability, and significant side effects, which prevent them from acting specifically on inflammatory sites and balancing excessive ROS accumulation and intestinal barrier damage. UC inflamed regions feature elevated myeloperoxidase (MPO) expression and enriched CD44^+^ receptors on macrophages, while excess ROS worsens epithelial barrier disruption and inflammatory cytokine release. Though CeO_2_ NPs efficiently scavenge ROS, their inadequate targeting causes systemic exposure. Hyaluronic acid (HA) specifically binds CD44^+^ receptors, and serotonin (5-HT) targets MPO, with their synergy compensating for individual limitations. Accordingly, researchers developed a novel targeted UC nanomedicine: hyaluronic acid/5-hydroxytryptamine-modified CeO_2_ NPs (HA-5-HT@CeO_2_). Studies show HA-5-HT@CeO_2_ excels at scavenging ROS and inflammatory mediators, repairing the intestinal epithelial barrier, and alleviating colitis symptoms. It exhibited good biocompatibility with no significant toxicity in both *in vitro* cell and *in vivo* animal experiments, holding high clinical application potential 43.

The treatment of IBD still faces numerous bottlenecks. Existing biologics are associated with high costs, immunogenicity, and side effect risks. JAK inhibitors are also limited by contraindications in specific populations. Approximately 30% of patients do not respond to current therapies, urgently requiring the development of novel treatment options with unique mechanisms and convenient delivery. As a key anti-inflammatory molecule, miR146a suppresses NF-κB activation by targeting IRAK1 and TRAF6, reducing pro-inflammatory factor release, but its RNA nature renders it vulnerable to gastrointestinal acid and enzymatic degradation, hindering oral delivery. CeO_2_ NPs exhibit excellent ROS-scavenging capacity, biocompatibility, and serve as stable biomolecule carriers. To address these challenges, Apte *et al*. 44 constructed an oral nanoparticle formulation (CNP-miR146a) by chemically conjugating miR146a with CeO_2_ NPs, resolving miR146a's gastrointestinal stability issues while leveraging synergistic antioxidant (CeO_2_ NPs) and anti-inflammatory (miR146a) effects. The study first validated CNP-miR146a's stability in simulated gastrointestinal environments, showing resistance to degradation and significant therapeutic efficacy in a mouse model of chronic colitis. Results demonstrated reduced colitis symptoms: diminished inflammatory cell infiltration, lower oxidative stress marker 8-OHdG levels, and decreased pro-inflammatory cytokines (IL-6, TNF). Though no clinical trials were conducted, CNP-miR146a's oral stability, notable *in vivo* therapeutic effects, and novel mechanism support its potential for clinical trials, offering a new IBD treatment strategy and insights for oral RNA drug delivery.

#### 2.3.2. Osteoarthritis (OA)

OA, characterized by chronic inflammation and cartilage degeneration, severely impacts the lives of hundreds of millions of people worldwide. Current treatments primarily focus on symptom management rather than addressing the underlying disease mechanisms. Research on functionalized CeO_2_ NPs offers new avenues for developing nanotherapeutics targeting inflammatory joint diseases.

The core pathological mechanism of OA is closely associated with oxidative stress induced by excessive ROS and reactive nitrogen species (RNS), as well as abnormal activation of the ROS/Rac-1/NF-κB pathway. This leads to chondrocyte apoptosis, release of inflammatory mediators, and degradation of the extracellular matrix, while existing therapeutic approaches struggle to precisely regulate the inflammatory microenvironment. Traditional CeO_2_ NPs possess SOD/CAT-like enzymatic activity but suffer from limited oxygen vacancy density and insufficient catalytic efficiency, restricting their therapeutic potential. To address this, researchers employed a defect engineering strategy by co-doping copper (Cu) and platinum (Pt): Cu increases the Ce^3+^/Ce^4+^ ratio to boost oxygen vacancy density, while Pt stabilizes the more active Cu^+^ and forms strong metal-carrier interactions with CeO_2_. Their combined light absorption properties confer photothermal responsiveness to the material, with NIR irradiation further enhancing enzymatic catalytic activity. Based on this, Yang *et al*. 13 designed a novel oxygen vacancy-engineered ceria nanozyme (PtCuO_x_/CeO_2-x_) through co-doping copper (Cu) and platinum (Pt). Experimental results demonstrate that PtCuO_x_/CeO_2-x_ efficiently scavenges IL-1β-induced ROS/RNS within chondrocytes, protects mitochondrial function (restoring membrane potential and adenosine triphosphate (ATP) production), inhibits chondrocyte apoptosis, downregulated proinflammatory factors and matrix degrading enzymes, and upregulated chondroprotective factors. In the ACLT rat OA model, PtCuO_x_/CeO_2-x_ + NIR therapy significantly repaired cartilage damage and improved rat gait function. Immunohistochemistry confirmed its ability to inhibit Rac-1 and p-p65 expression, blocking the ROS/Rac-1/NF-κB pathway. Leveraging its core advantages of “high-efficiency SOD/CAT-like activity, photothermal responsiveness, and excellent biosafety”, PtCuO_x_/CeO_2-x_ demonstrates clear clinical translation potential in treating ROS-mediated inflammatory diseases, particularly in the field of OA.

As a typical multi-stage degenerative disease, the pathological process of OA encompasses an initial phase of intense inflammatory response followed by a subsequent stage of cartilage repair and proliferation. Current monotherapies with single nanoparticles only target specific stages, limiting full-course intervention and therapeutic efficacy. Collagen hydrogels, biocompatible and injectable with cartilage extracellular matrix-mimicking properties, are ideal nanomedicine carriers. Researchers have developed a thiol-crosslinked collagen hydrogel (CSH-CeO_2_-pFe_2_O_3_) by leveraging the antioxidant and anti-inflammatory properties of CeO_2_ NPs and the chondrogenic effects of Fe_2_O_3_ nanoparticles (Fe_2_O_3_ NPs). CeO_2_ NPs are directly embedded in the hydrogel matrix, while Fe_2_O_3_ NPs are encapsulated in PLGA microspheres before loading, with a ROS-responsive release mechanism enabling "rapid anti-inflammation in the inflammatory phase + sustained regeneration promotion in the proliferative phase" to address OA's pathological progression comprehensively. Experimental results confirm CSH-CeO_2_-pFe_2_O_3_ has excellent mechanical strength, injectability, and biocompatibility, significantly enhancing cell adhesion, proliferation, and chondrogenic differentiation. It intelligently responds to high ROS levels by rapidly releasing CeO_2_ NPs to alleviate inflammation, followed by sustained Fe_2_O_3_ release for cartilage regeneration, improving treatment precision and effectiveness with remarkable therapeutic outcomes. This hydrogel provides a novel strategy for clinical OA treatment, promising more effective pain and inflammation relief via local sustained drug release 45.

#### 2.3.3. Endometriosis

Endometriosis is characterized by ectopic implantation and growth of endometrial tissue, chronic inflammatory activation, and oxidative stress overload, severely impacting reproductive health and quality of life in women of childbearing age. Current treatments primarily rely on hormonal interventions or surgical excision, which can temporarily alleviate pain and disease progression but are limited by significant side effects and high recurrence rates, failing to target the fundamental mechanisms of the disease. The anti-inflammatory properties, ROS scavenging capabilities, and targeted delivery characteristics of functionalized CeO_2_ NPs open new avenues for developing precision nanotherapeutic drugs for endometriosis.

Rahman *et al*. 46 developed CeO_2_ NPs as a non-steroidal anti-inflammatory agent for endometriosis therapy. Synthesized via biomineralization on a bovine serum albumin (BSA) substrate, the CeO_2_ NPs possess antioxidant and enzymatic activities. Benefiting from albumin's properties, they passively target inflammatory sites (especially ectopic lesions) while sparing healthy tissues. Conjugated with near-infrared fluorescent dye ICG, CeO_2_ NPs enable non-invasive imaging for real-time tracking of drug distribution, aiding clinical applications. *In vitro*, CeO_2_ NPs reduced M1 macrophage marker CD80 expression and increased M2 marker ARG1 expression, demonstrating potent anti-inflammatory effects. In a mouse endometriosis model induced by autologous uterine tissue transplantation, intravenous administration of CeO_2_ NPs (120 μg/kg) was evaluated via fluorescent/photoacoustic imaging (for lesion accumulation) and histological analysis. Results showed significant reduction in ectopic lesion numbers without impairing early pregnancy (implantation/decidualization). As CeO_2_ NPs passively target ectopic lesions and allow real-time tracking via non-invasive imaging without disrupting normal pregnancy, this nonsteroidal agent integrates anti-inflammatory therapy with lesion imaging, offering a novel “precision treatment + real-time monitoring” theranostic approach for endometriosis with substantial clinical translation potential.

#### 2.3.4. Acute Lung Injury (ALI)

ALI is characterized by acute inflammatory bursts in the lungs, disruption of the pulmonary epithelial/endothelial barrier, and excessive oxidative stress, posing a severe threat to the lives and prognosis of critically ill patients. Current treatments primarily focus on respiratory support and broad-spectrum anti-inflammatory therapies, emphasizing symptom relief while failing to target the fundamental pathophysiological mechanisms involving the interplay of inflammation and oxidative stress. The anti-inflammatory, antioxidant, and tissue-protective properties of functionalized CeO_2_ NPs open new avenues for developing precision nanotherapeutic agents specifically for acute lung injury.

Polydopamine (PDA) serves as a biocompatible material that combines NIR photothermal conversion capability with ROS scavenging activity. Its phenolic hydroxyl groups enhance ROS removal, while its photothermal effect can modulate nanozyme catalytic activity, thereby addressing the limitations of CeO_2_ in terms of limited catalytic efficiency and lack of synergistic enhancement mechanisms. Based on this, Yin *et al*. 47 developed a novel PDA-coated ceria nanoenzyme (Ce@P), whose anti-inflammatory efficacy arises from PDA-CeO_2_ synergy, further amplified by NIR irradiation. The reversible Ce^3+^/Ce^4+^ conversion in CeO_2_ cooperates with PDA's phenolic hydroxyl groups to scavenge excess pulmonary ROS (O_2_^-^, ·OH, H_2_O_2_) and suppress ROS-mediated inflammatory signaling (e.g., NF-κB activation). In LPS-induced RAW264.7 macrophages, PDA and CeO_2_ jointly downregulated pro-inflammatory factor transcription, induced M2 macrophage polarization, and reduced inflammatory cell infiltration. Such anti-inflammatory effects can be further enhanced by NIR irradiation. NIR not only boosts Ce@P's ROS scavenging efficiency but also promotes heat shock protein gene (HSP70) expression via thermal activation, strengthening anti-inflammatory and tissue repair capacities. Notably, Ce@P exhibits excellent biocompatibility, causing no inflammatory damage to non-pulmonary organs (heart, liver, spleen, kidney). After intravenous injection, it significantly enriches in the lungs and degrades gradually. Endowed with targeted enrichment, ROS scavenging, anti-inflammatory effects, photothermal synergy, and favorable biosafety, Ce@P holds promising prospects for ALI treatment.

#### 2.3.5. Age-related Macular Degeneration (AMD)

AMD is characterized by chronic oxidative stress, inflammatory damage, and photoreceptor degeneration in the macular region. It poses a severe threat to the visual health of tens of millions of elderly individuals worldwide and can even lead to irreversible blindness. Current treatments lack effective interventions for dry AMD, while wet AMD relies on anti-vascular endothelial growth factor drugs to mitigate vascular proliferation. Neither therapeutic strategy targets the fundamental disease mechanisms driven by oxidative stress and inflammation. Functionalized CeO_2_ NPs, with their efficient ROS scavenging, targeted anti-inflammatory effects, and excellent biocompatibility, open new avenues for developing precision nanotherapeutics against AMD.

**Figure 4:**
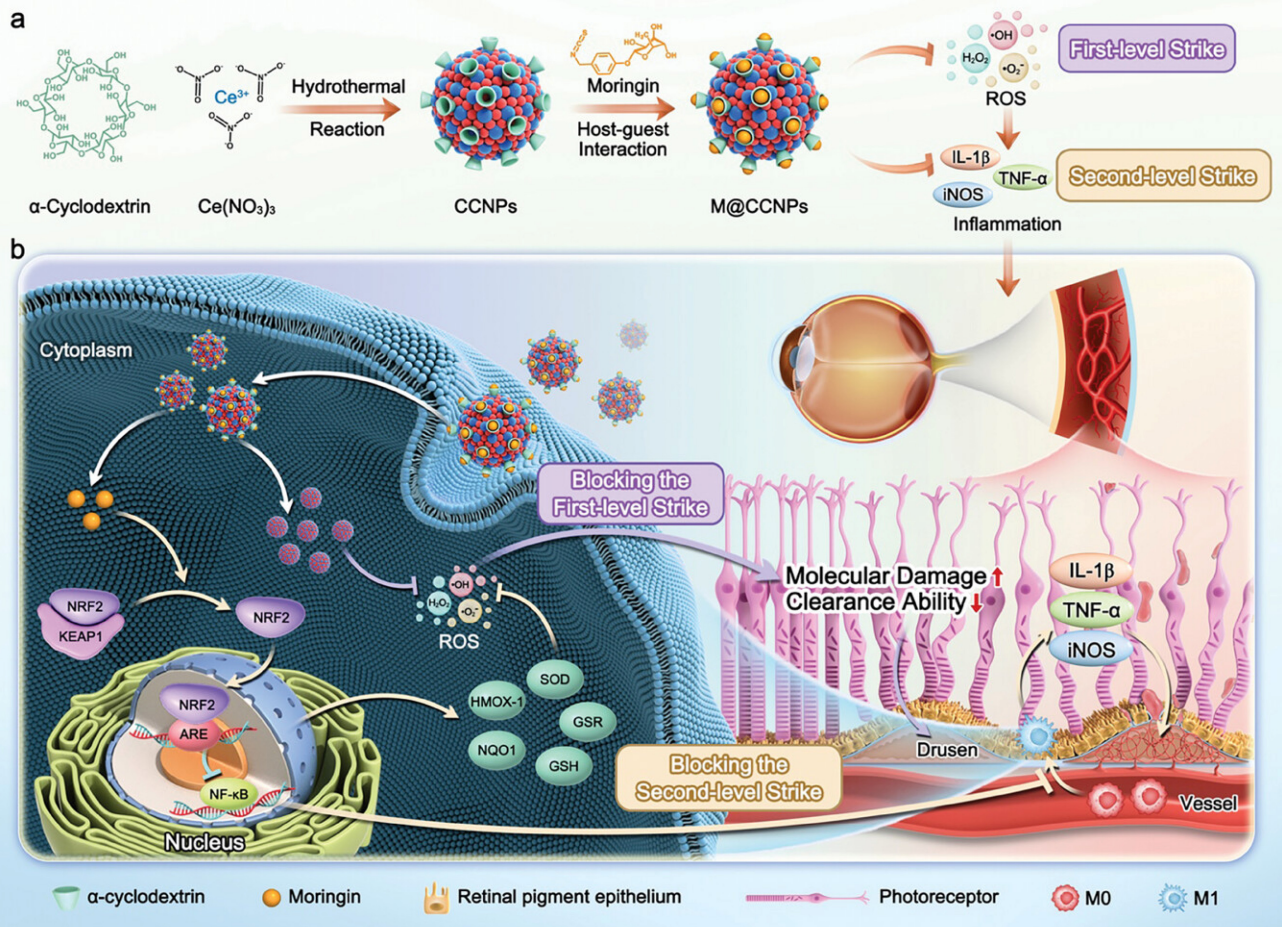
Schematic illustration of M@CCNP for management and amelioration of AMD via blocking the two-level strike. a) The synthesis of M@CCNP by one-pot hydrothermal method for CCNP preparation and host-guest interaction for MOR loading. b) The underlying therapeutic mechanism of M@CCNP via its unique properties of auto-regenerative ROS scavenging and inflammation suppression, thus blocking the two-level pathological response of AMD. Reprinted from Ref. 48. Open-access article. Permission not required.

The pathogenesis of AMD follows a “two-stage” model: Stage I involves excessive oxidative stress damaging retinal pigment epithelial cells and forming drusen; Stage II triggers inflammatory responses. These stages mutually exacerbate, activate downstream vascular endothelial growth factor (VEGF) signaling, and ultimately lead to choroidal neovascularization (CNV). Single-target therapies cannot fully intervene, creating an urgent need for integrated platforms blocking both stages. CeO_2_ NPs, with reversible Ce^3+^/Ce^4+^ conversion for renewable ROS scavenging, inhibits the first stage. Moringin (MOR) exerts potent anti-inflammatory effects via NRF2 pathway activation but suffers from poor solubility due to hydrophobicity. As a biocompatible carrier, α-cyclodextrin (α-CD) enhances the biocompatibility of CeO_2_ NPs through O-Ce bond encapsulation and improves the solubility of MOR via host-guest interactions. As shown in **Figure 4**, Xu *et al*. 48 constructed MOR-loaded α-CD-coated CeO_2_ NPs (M@CCNP) via supramolecular engineering. This nanotechnology platform combines the self-regenerating antioxidant capacity of CeO_2_ NPs with the anti-inflammatory properties of MOR, enabling dual inhibition of oxidative stress and inflammatory pathways. *In vitro*, M@CCNP significantly inhibited LPS-induced ROS production and inflammation, with good biocompatibility and cellular uptake. In a mouse CNV model, it remarkably reduced CNV lesions, decreased leakage area, and restored fundus structure integrity. This treatment showed superior efficacy in reducing leakage compared with aflibercept in the experimental model, a traditional anti-VEGF agent. Surface modification endows CeO_2_ NPs with distinct advantages in drug loading and targeting, further emphasizing their great potential as versatile delivery platforms.

#### 2.3.6. Dry Eye Disease (DED)

Dry eye disease is a common ocular condition affecting a substantial proportion of the global population. Current treatments, such as artificial tears, have limitations including long-term side effects and slow onset of action, necessitating the development of novel therapeutics with antioxidant and anti-inflammatory properties.

HA, as an endogenous biocompatible biomolecule, improves the thickness and stability of the tear film while exerting a moisturizing effect on the ocular surface. Meanwhile, CeO_2_ acts as a highly efficient ROS scavenger by virtue of the reversible redox conversion between Ce^3+^ and Ce^4+^ valence states. Leveraging their complementary advantages, Wu *et al*. 49 developed a novel nanomaterial (HA-CeO_2_) based on HA-modified CeO_2_ for the treatment of DED. *In vitro* studies showed that HA-CeO_2_ exhibited remarkable antioxidant and anti-inflammatory effects, efficiently scavenging ROS, alleviating oxidative stress, and downregulating the expression of inflammatory factors. In a dry eye mouse model, HA-CeO_2_ significantly improved corneal epithelial damage, tear secretion, and tear film stability, indicating promising therapeutic potential.

### 2.4. Wound Healing

#### 2.4.1. Corneal Abrasion (CA)

CA are common ocular surface injuries, with core pathologies involving corneal epithelial damage, oxidative stress, and inflammatory responses. Without timely and effective intervention, they may progress to severe corneal pathologies. Current treatments primarily rely on analgesics like ketorolac, which alleviate pain but fail to promote wound healing. Prolonged use may induce side effects such as tissue ulceration.

β-1,3-glucan, a natural wound-healing promoter that regulates cell migration and proliferation, is limited by low bioavailability and short corneal retention, with the latter attributable to the ocular blink reflex and tear drainage. CeO_2_ NPs have attracted attention for ocular surface disease treatment owing to their antioxidant activity (from reversible valence conversion) and porous structure enabling drug loading, but pure CeO_2_ suffers from poor mucoadhesion and insufficient cellular uptake, restricting therapeutic efficacy. Alginate (ALG), a biocompatible natural polysaccharide, enhances mucoadhesion and prolongs corneal retention via hydrogen bonding. It also has unique ion-responsive properties: forming stable "egg-crate structures" with Ca^2+^ for efficient drug encapsulation and undergoing ion exchange with tear Na^+^ for sustained drug release, while improving nanoparticle cellular uptake. Based on this, Ger *et al*. 50 developed an alginate-functionalized CeO_2_ NP (Ce-ALG) eye drop formulation for CA treatment. *In vitro* experiments confirmed Ce-ALG's good biocompatibility and enhanced antioxidant, anti-inflammatory, and anti-apoptotic activities. In a rabbit CA model, Ce-ALG significantly reduced corneal damage area, accelerated epithelial recovery, and inhibited inflammation. Thus, Ce-ALG holds promise as an effective drug carrier for corneal injury treatment.

#### 2.4.2. Soft Tissue Repair

CeO_2_ NPs demonstrate remarkable efficacy and favorable biocompatibility in the wound healing process. Their multiple mechanisms of action include antioxidant properties, antibacterial effects, and the promotion of wound healing. It can not only effectively clear ROS in the wound, alleviate oxidative stress, but also exert antibacterial effects by catalyzing the production of hydroxyl radicals, and promote cell proliferation and migration by regulating the wound microenvironment, accelerating wound healing. In addition, CeO_2_ NPs can also be used in combination with other bioactive ingredients such as Bletilla striata polysaccharide (BSP) and epidermal growth factor (EGF) to get the synergistic effect and further improve the effect of wound healing 51.

A novel multifunctional hydrogel (Pltm@CeO_2_ NPs/Gel) was developed, which embeds platelet membrane-camouflaged CeO_2_ NPs in a gelatin methacrylate (GelMA) matrix to accelerate diabetic wound healing. CeO_2_ NPs possess the capability to scavenge ROS, which can mitigate oxidative stress and inflammatory responses at wound sites, thereby establishing a conducive microenvironment for wound healing. Using platelet membrane (PLTm) to encapsulate CeO_2_ NPs simulates the function of platelets, promotes platelet aggregation and activation at the wound site, and accelerates blood coagulation and angiogenesis. At the same time, the GelMA hydrogel provides a suitable microenvironment for wound healing, which can absorb exudate and maintain moisture. This study integrates antioxidant, anti-inflammatory, and pro-angiogenic functions into a hydrogel system, providing a comprehensive solution for diabetic wound healing 52.

#### 2.4.3. Bone Tissue Regeneration

Bone tissue defect repair presents a significant challenge in clinical orthopedics. Bone defects caused by trauma, tumor resection, congenital deformities, and other factors often fail to achieve ideal repair due to limited regenerative capacity or issues with traditional treatments (such as autologous bone grafting and allogeneic bone grafting), including donor shortages, immune rejection, and infection risks. The rapid advancement of nanomaterials has brought new breakthroughs to bone tissue regeneration. Among these, CeO_2_ NPs have emerged as highly promising regenerative medical materials due to their unique redox activity, excellent biocompatibility, and low cytotoxicity.

Considering bioactive glass (BG) as a classic bone repair material, while it can stimulate bone matrix formation by releasing calcium and silicon ions, its inherent brittleness and lack of antioxidant and anti-inflammatory activity make it difficult to address the vicious cycle of oxidative stress and inflammatory responses in the early stages of bone defects. 3D printing enables precise fabrication of porous scaffolds matching defect morphology to support cell infiltration and new bone growth, while CeO_2_ NPs efficiently scavenge ROS, regulate inflammation, and promote osteoblast proliferation/differentiation. Zhang *et al*. 53 developed a multifunctional 3D-printed scaffold by integrating CeO_2_ NPs into BG via ball milling (CeO_2_-BG), combining CeO_2_'s antioxidant/osteogenic properties with BG's structural support for sequential inflammation management and osteogenesis. This modification enhanced BG's mechanical strength (from 2.17 MPa for pure BG to 34.24 MPa for CeO_2_-BG-20%) and endowed it with antioxidant, anti-inflammatory, and osteogenic capabilities. *In vitro*, CeO_2_-BG promoted rat osteoblast proliferation and osteogenic differentiation by increasing mineral deposition, alkaline phosphatase (ALP) activity, and osteogenic gene expression, while efficiently scavenging excess ROS to reduce oxidative stress. In a rat tibial defect model, micro-CT and histological analysis showed significantly higher new bone formation in the CeO_2_-BG-20% group than in pure BG and blank groups, with superior bone volume/tissue volume (BV/TV), trabecular thickness (Tb.Th), and trabecular number (Tb.N). Thus, CeO_2_-functionalized BG scaffolds offer innovative potential for clinical bone defect repair as next-generation bone regeneration materials.

Bone defect repair faces key challenges: inflammatory microenvironment interference, low stem cell survival post-transplantation, and insufficient osteogenic induction. Inflammatory responses at defect sites drive macrophage M1 polarization, releasing excessive inflammatory mediators that inhibit bone marrow-derived mesenchymal stem cell (BMSC) proliferation and osteogenic differentiation. Traditional gelatin methacrylate (GelMA) hydrogels, while mimicking the extracellular matrix (ECM) and loading BMSCs, lack mechanical strength, anti-inflammatory activity, and osteogenic capacity, failing to meet complex bone repair demands. CeO_2_ NPs, with unique antioxidant properties, immunomodulatory functions (inhibiting M1 and promoting M2 polarization), and osteogenic effects, address GelMA's limitations. Wang *et al*. 54 constructed a GelMA-CeO_2_-BMSC composite hydrogel by combining CeO_2_ NPs, GelMA, and BMSCs. *In vitro*, it showed superior osteoinductive and immunomodulatory properties compared with traditional GelMA hydrogels by regulating macrophage polarization to alleviate inflammation and construct a favorable microenvironment for BMSC osteogenic differentiation, which is a critical mechanism for bone repair. Both *in vitro* and *in vivo* experiments confirmed its significant bone defect repair efficacy, highlighting promising application prospects.

Periodontal bone defects from periodontitis are a key challenge in periodontal regenerative therapy. Existing guided tissue/bone regeneration (GTR/GBR) membranes prevent fibrous tissue invasion but suffer from insufficient mechanical strength, inadequate osteogenic induction, and improper degradation rates, failing to meet complex regeneration demands. Electrospun fiber membranes mimic the natural ECM's porous structure, serving as ideal tissue engineering scaffolds. The PCL-gelatin composite system offers biocompatibility and degradability but lacks active osteogenic properties. CeO_2_ NPs possess excellent biocompatibility, stem cell osteogenic differentiation-promoting capacity, and antioxidant activity, which enables them to mitigate this deficiency. Nevertheless, their poor hydrophilicity limits their application in the biomedical field. To resolve this, Ren *et al*. 55 synthesized CeO_2_ NPs via chemical methods and modified them with citric acid to enhance hydrophilicity. They fabricated a PG-CeO_2_ composite fiber membrane by loading these NPs into PCL-gelatin via electrospinning. This membrane features an ECM-mimicking porous structure that facilitates cell migration, adhesion, proliferation, and differentiation, with good mechanical properties and sustained CeO_2_ NPs release for continuous bioactive support. Results confirmed CeO_2_ NPs' biocompatibility and ability to promote human periodontal ligament stem cell (hPDLSC) proliferation and osteogenic differentiation. PG-CeO_2_ fiber membranes exhibited excellent bone regeneration capacity both *in vitro* and *in vivo*, emerging as a promising novel biomaterial for periodontal bone regeneration.

### 2.5. Neuroprotection

#### 2.5.1. Central Nervous System (CNS)

CNS-related neurodegenerative diseases (such as Alzheimer's disease and Parkinson's disease) are characterized by chronic inflammation, oxidative stress-mediated neuronal damage, and synaptic dysfunction. These conditions severely threaten the cognitive and motor functions of tens of millions of people worldwide, often leading to irreversible functional loss. Current treatments primarily alleviate symptoms symptomatically, failing to interrupt the fundamental disease mechanism driven synergistically by inflammation and oxidative stress. Functionalized CeO_2_ NPs, with their targeted antioxidant, anti-inflammatory, and neuroprotective properties, open new avenues for developing precision nanotherapeutic drugs targeting CNS inflammation-related diseases.

CNS inflammation is a core pathological mechanism in neurodegenerative diseases and post-brain injury conditions, driven primarily by excessive microglial activation. LPS and other stimuli trigger microglia to secrete proinflammatory factors (TNF-α, IL-1β), ROS, RNS and NO, which activate the FAK/STAT3 signaling pathway downstream of integrin αvβ3, forming a detrimental "inflammation-oxidative stress" cycle that aggravates neuronal damage. Conventional monotherapies targeting single mechanisms fail to comprehensively block this pathological process. Jia *et al*. 56 synthesized CeO_2_@PAA nanoparticles by modifying CeO_2_ with PAA, and further prepared CeO_2_@PAA-LXW7 composites via EDC-mediated covalent conjugation of biotinylated RGD cyclic peptide LXW7 to the carboxyl groups on PAA. LXW7, a peptide that specifically binds integrin αvβ3, synergizes with CeO_2_@PAA to strengthen the inhibition of integrin signaling. *In vitro* studies showed that CeO_2_@PAA-LXW7 potently suppressed LPS-induced BV2 microglial activation, reduced the production of TNF-α, IL-1β and other proinflammatory cytokines, and inhibited the release of NO and ROS. It also significantly downregulated integrin αvβ3 expression and the activation of its downstream FAK/STAT3 pathway (including the phosphorylation of FAK and STAT3) in LPS-stimulated BV2 cells. By alleviating neuroinflammation and oxidative stress, CeO_2_@PAA-LXW7 exerts robust neuroprotective effects, offering novel targets and mechanisms for neuroinflammation treatment. This composite thus holds promise as an inhibitor of CNS inflammation, warranting further *in vivo* validation in animal models of neurodegenerative diseases or brain injury.

Following spinal cord injury (SCI), the CNS struggles to regenerate spontaneously. A series of secondary injuries, including uncontrolled inflammation, oxidative stress and glial scar formation, aggravate neuronal death and axonal damage, which ultimately induces motor function loss and intractable neuropathic pain. Existing treatments struggle to simultaneously address the dual demands of structural repair and pathological microenvironment regulation. PCL composite nanofiber scaffolds, mimicking ECM architecture with excellent biocompatibility and degradability, emerge as ideal structural support carriers for SCI repair. However, standalone scaffolds lack antioxidant and anti-inflammatory activity, failing to interrupt the vicious cycle of secondary injury. CeO_2_ NPs combine potent ROS scavenging capacity with anti-inflammatory activity, suppressing microglial activation (reducing Iba-1 expression) and alleviating neuroinflammation while promoting neuronal survival and axonal stability. Rahimi *et al*. 57 compounded CeO_2_ NPs with gelatin and stably integrated them onto the surface of gelatin-PCL nanofiber scaffolds using electrospray technique. The incorporation of CeO_2_ NPs into the scaffold was aimed at leveraging their antioxidant and anti-inflammatory properties to promote nerve regeneration and alleviate pain. The results showed that the nanofiber scaffolds significantly improve motor function and pain symptoms after spinal cord injury, providing a new strategy for the treatment of spinal cord injury.

Alzheimer's disease (AD) is characterized by a vicious cycle of Aβ aggregation and oxidative stress, with excessive ROS exacerbating neuronal damage and the blood-brain barrier (BBB) hindering drug delivery. Current therapies fail to simultaneously address Aβ clearance, oxidative stress regulation, and BBB penetration. Resveratrol (RES) has anti-inflammatory, antioxidant, and Aβ aggregation-inhibiting properties but is limited by poor water solubility, low bioavailability, and inability to cross the BBB. CeO_2_ NPs scavenge ROS but lack sufficient catalytic activity and targeting. To overcome these drawbacks, Hu *et al*. 58 synthesized manganese-doped CeO_2_ hollow nanoparticles (LMC) and loaded them with RES to form LMC-RES, enhancing RES's solubility and bioavailability. Manganese doping and lactoferrin modification improved the nanoparticles' catalytic activity, biocompatibility, and BBB penetration, enabling LMC-RES to reach and act on brain neurons and microglia. *In vitro*, LMC-RES mitigated Aβ-induced oxidative stress via the Nrf-2/HO-1 pathway, reducing ROS and protecting neural cells. In AD model mice, it lowered ROS levels, inhibited Aβ aggregation, protected neurons, and significantly ameliorated cognitive dysfunction.

Aggregation of β-amyloid (Aβ1-42), abnormal aggregation of tau protein forming neurofibrillary tangles, and cholinergic dysfunction caused by abnormally elevated acetylcholinesterase (AChE) and butyrylcholinesterase (BuChE) activity make it difficult for single-target therapies to halt the progression of AD. Existing drugs like donepezil inhibit cholinesterases but show no significant effect on protein aggregation and carry notable side effects. Natural products geniposide and harpagoside exhibit potential for neuroprotection and anti-inflammatory effects, yet suffer from poor water solubility, low bioavailability, and limited BBB penetration. CeO_2_ NPs, with their excellent antioxidant properties and drug-loading capacity, can serve as delivery vehicles for natural compounds. However, when used alone, they have only a limited inhibitory effect on protein aggregation. Pérez Gutiérrez *et al*. 59 synthesized CeO_2_ NPs via the sol-gel method and combined them with geniposide and harpagoside to form GH/CeO_2_ NPs. Encapsulation of GH within CeO_2_ NPs enhanced nanoparticle stability, biocompatibility, and brain delivery. The fabrication of GH/CeO_2_ nanoparticles enhanced the water solubility and bioavailability of GH, rendering it more appropriates for neuroprotective applications. GH/CeO_2_ NPs not only suppressed the aggregation of Aβ1-42 and Tau proteins but also improved cognitive function by inhibiting AChE and BuChE, illustrating multi-target therapeutic potential. The study revealed the mechanism of neuroprotection by GH/CeO_2_ NPs through the inhibition of protein aggregation and cholinesterase activity, providing a theoretical basis for future drug development.

Acute cerebral ischemia-reperfusion injury is a major challenge post-thrombolytic therapy for ischemic stroke. Reperfusion triggers an oxidative stress surge in the ischemic penumbra, with excessive ROS directly inducing neuronal apoptosis and disrupting BBB integrity, exacerbating cerebral edema and brain damage. Conventional thrombolytics only restore vascular patency without blocking this pathological process. CeO_2_ NPs exhibit potent ROS-scavenging activity but lack brain targeting, leading to systemic distribution and insufficient brain accumulation that limits efficacy. Integrin αvβ3 is selectively overexpressed in ischemic brain regions during reperfusion, serving as an ideal target for site-specific delivery. LXW7, a high-affinity integrin αvβ3 inhibitor, binds specifically to this target and has a structure suitable for coupling with nanoparticles. Zhang *et al*. 60 synthesized bLXW7-CeNP by conjugating LXW7 with CeO_2_ NPs. Via LXW7-integrin αvβ3 binding, CeO_2_ NPs were efficiently targeted to the ischemic penumbra, enhancing local antioxidant capacity and alleviating ischemia-reperfusion injury. Experiments showed both bLXW7-CeNP and CeO_2_ NPs improved neurological deficit scores (no significant intergroup difference), but the bLXW7-CeNP group had significantly smaller infarct volumes than the CeO_2_ NPs and control groups, with enhanced neuroprotection. Additionally, bLXW7-CeNP better maintained BBB integrity (less disruption vs. CeO_2_ NPs and control groups), showed superior oxidative stress indicators (GSH/GSSG ratio and SOD activity), and reduced expression of apoptotic protein cleaved caspase-3, demonstrating stronger antioxidant and anti-apoptotic effects. This study provides experimental evidence for developing novel cerebral ischemia therapies, potentially overcoming limitations of existing thrombolytics and reducing post-ischemic neuronal damage.

CeO_2_ NPs hold promises as neuroprotective agents, yet their biological activity remains highly dependent on surface stabilizers. Stabilization with citric acid (CA) alone may induce pro-oxidative effects, while EDTA alone may compromise colloidal stability, making it difficult to simultaneously optimize enzyme activity, stability, and biocompatibility. To overcome this challenge, optimizing stabilizer combinations is essential to regulate the surface properties of CeO_2_ NPs, balancing their antioxidant activity with *in vivo* behavior. Based on this, Estevez *et al*. 61 focused on regulating the ratio of CA to EDTA, systematically investigating the effects of different ratios on the enzyme-mimetic activity, stability, and neuroprotective efficacy of CeO_2_ NPs. Their aim was to identify an optimal formulation combining high antioxidant efficiency with good biocompatibility, offering a novel approach for treating ischemic brain injury. They synthesized CeO_2_ NPs by varying the ratio of CA and EDTA, revealing that different CA/EDTA ratios significantly impact the antioxidant enzyme-mimicking activity (including CAT, SOD, and oxidase activity) and neuroprotective effects of CeO_2_ NPs. Specifically, CeO_2_ NPs with equal proportions of CA/EDTA (50/50) exhibited the best antioxidant and neuroprotective effects, significantly reducing cell death and oxidative stress induced by ischemia/reperfusion injury. These CeO_2_ NPs also demonstrated good bioavailability *in vivo*, accumulating and maintaining activity in brain tissue. Therefore, CeNPs exhibit solid potential for clinical translation in oxidative stress-related neurological diseases.

A composite carrier composed of PEG and poly(lactic-co-glycolic acid) (PLGA) exhibits excellent biocompatibility, prolonged circulation properties, and controlled release capabilities. PEG reduces clearance of nanoparticles by the reticuloendothelial system, while PLGA protects CeO_2_ and enables sustained release. Their synergistic action significantly enhances the BBB penetration efficiency and targeting of nanomaterials. Gao *et al*. 62 developed a nanocomposite by combining CeO_2_ with PEG and PLGA. This composite leverages the properties of PEG and PLGA to enhance the biocompatibility and targeting of the nanoparticles, enabling CeO_2_ to better penetrate the BBB and exert neuroprotective effects. In the middle cerebral artery occlusion (MCAO) model, the CeO_2_-PEG/PLGA nanocomposite significantly reduced infarct volume and brain edema, demonstrating promising neuroprotective effects and offering a novel strategy for cerebral ischemia treatment.

The BBB impedes targeted drug delivery in Alzheimer's disease therapy. Natural bioactive compounds like curcumin (Cur) require high concentrations to reverse the glial cell phenotype, yet suffer from poor water solubility and insufficient local concentrations. While CeO_2_ NPs can scavenge ROS and depolymerize Aβ, they lack precise targeting and controlled release mechanisms, hindering multi-target synergistic therapy. To overcome these challenges, a delivery system must be developed that combines BBB penetration capability, responsive release within the lesion microenvironment, and multi-effect synergistic functionality. Han *et al*. 63 selected a polymer with thermosensitive deformation properties (pNIPAAm-co-pAAm). At temperatures above the lower critical solution temperature (LCST), the polymer can be compressed to encapsulate hydrophobic drugs (such as Cur) and fixed on ultra-small CeO_2_ NPs through elastic compression. A dopamine self-polymerization shell was formed to further encapsulate the drug, and apolipoprotein (apoA-I) modification was used to enhance brain transport. The dopamine shell undergoes degradation triggered by ROS, leading to expansion of the internal polymer structure and restoration of hydrophilicity, which enables rapid release of Cur and complete exposure of CeO_2_. This polymer-based biomimetic assembly can respond to ROS in the brain by undergoing elastic expansion and drug release. The rapid release of Cur can concentration-dependently convert Aβ-activated microglia into normal microglia, reducing the secretion of inflammatory factors, increasing the secretion of anti-inflammatory factors, and promoting the uptake and degradation of Aβ. CeO_2_ NPs can depolymerise Aβ fibres, reduce Aβ deposition, and promote the uptake and degradation of Aβ by microglia. In AD mouse models, APPCeO_2_/Cur could significantly reduce Aβ deposition, inflammatory factor expression, and oxidative stress, and improve neuronal damage and cognitive ability.

#### 2.5.2. Peripheral Nervous System (PNS)

Peripheral neuropathy (PN) is characterized by chronic inflammation, oxidative stress-mediated nerve fiber damage, and myelin degeneration. It severely impairs sensory and motor function in hundreds of millions of patients worldwide, often accompanied by pain, numbness, and even limb disability. Current treatments primarily focus on symptom relief (e.g., pain management, nerve nutrition) without addressing the fundamental disease mechanism driven by the synergistic interaction of inflammation and oxidative stress. Functionalized CeO_2_ NPs, with their potent anti-inflammatory properties, targeted ROS scavenging, and neuroprotective effects, open new avenues for developing precision nanotherapeutics targeting inflammatory disorders of the peripheral nervous system.

Transient receptor potential (TRP) channels serve as key regulators of neuronal excitability and are activated by intracellular redox changes. Exogenous ROS induce Ca^2+^ influx by modifying channel cysteine residues to depolarize neurons, while excessive ROS trigger oxidative stress and neuronal damage. This process forms the core “activation-damage” paradox in ROS-mediated neuromodulation. To address this, Liu *et al*. 64 developed a hybrid nanosystem (CZPN) by coating CeO_2_ nanocrystals (with inherent antioxidant activity) with metalloporphyrin ZnTPyP, enabling reversible TRP channel modulation and neuronal excitability regulation via photo-induced electrochemical reactions and antioxidant synergy. Upon light irradiation, ZnTPyP transfers electrons to CeO_2_ (converting Ce^4+^ to Ce^3+^) and activates ambient O_2_ to produce ROS. The altered redox state activates TRP channels and induces neuronal depolarization, while the nanosystem's high Ce^3+^/Ce^4+^ ratio scavenges excess ROS, protecting neurons from oxidative damage. This safe, effective, and universal photo-induced neuromodulation strategy offers a versatile photoelectrochemical approach for peripheral nervous system regulation and applications.

### 2.6. Imaging Applications

#### 2.6.1. For CT-guided diagnosis and treatment of IBD

The clinical management of IBD faces a dual challenge of “limited therapeutic efficacy” and “insufficient monitoring methods.” Traditional medications (e.g., 5-aminosalicylic acid) merely alleviate symptoms without interrupting the vicious cycle of ROS-mediated intestinal mucosal damage. Among existing non-invasive diagnostics, iodinated CT contrast agents suffer from short intestinal retention time, poor targeting of inflammatory sites, and insufficient signal intensity, preventing dynamic monitoring of treatment response and leading to a disconnect between diagnosis and therapy.

Cerium (Ce) has a higher K-edge (40.4 keV) than iodine (33.2 keV), making it compatible with clinical CT energies and a promising theranostic carrier. However, pure CeO_2_ NPs aggregate and precipitate in the acidic, enzyme-rich gastrointestinal environment, limiting oral application. Inulin (IN) is a naturally degradable polysaccharide that resists gastric acid and undergoes colonic microbiota-specific degradation, a characteristic that confers colon-targeting capability. Additionally, it improves nanoparticle dispersion and biocompatibility via steric hindrance. Li *et al*. 65 developed orally administered inulin-modified CeO_2_ nanozymes (CeO_2_@IN NPs), integrating antioxidant therapy and CT imaging on a single platform. *In vitro*, CeO_2_@IN showed a linear correlation between CT values and concentration, outperforming iodine-based ioversol at 80-140 kV with high sensitivity/resolution even at 0.5-10 mg/mL. In healthy mice, oral CeO_2_@IN visualized the entire gastrointestinal tract within 5 minutes, with sustained imaging for 6 hours. In DSS-induced colitis mice, it significantly accumulated in inflamed colon regions (retaining signal for 12 hours), while ioversol was largely cleared within 6 hours. CT 3D reconstruction revealed diminished signal in inflamed areas with treatment progression, synchronized with tissue repair and inflammation resolution, achieving “treatment-monitoring integration.” With superior CT contrast efficiency, inflammation-targeted retention, and dynamic monitoring capabilities, CeO_2_@IN outperforms traditional iodine contrast agents, enabling precision imaging-guided therapy for IBD.

#### 2.6.2. For MRI/CT dual-modality imaging-guided tumor therapy

The clinical translation of nanocatalytic chemotherapy (NCDT) is constrained by insufficient endogenous H_2_O_2_ levels, inefficient nanocatalyst performance, and the non-targeted depletion of H_2_O_2_ by CAT and GSH within the TME. Additionally, the lack of real-time visualization methods to precisely track treatment progression results in suboptimal efficacy and difficulty in therapeutic regulation. Dong S *et al*. 66 employed the Kirkendall effect to transform solid CeO_2_ into a hollow/ yolk-shell mesoporous structure. Co-doped with Mn^2+^/Zr^4+^, loaded with 3-amino-1,2,4-triazole (3-AT), and covalently modified with PEG-NH_2_, they constructed a CeO_2_ nanocatalyst platform (PHMZCO-AT) featuring precise multi-enzyme activity regulation, dual MRI/CT imaging, and TME responsiveness. Under TME conditions (pH 5.5), Mn^2+^ release increases, raising the r₁ value from 0.30 mM^-1^s^-1^ (pH 7.4) to 1.41 mM^-1^s^-1^ (pH 5.5), achieving tumor-specific signal enhancement. Leveraging Zr's high atomic number (Zr: 40) and strong X-ray attenuation properties enables high-resolution CT imaging. PHMZCO-AT achieves pH-responsive T1-MRI through Mn^2+^ and high-contrast CT imaging via Zr^4+^. Their synergy enables tumor-targeted localization and visual monitoring of therapeutic processes, providing a model for image-guided precision catalytic therapy.

#### 2.6.3. For CT and photothermal dual-modality imaging-guided colorectal cancer theranostics

The early diagnosis and precision treatment of colorectal cancer face dual challenges: early symptoms are often subtle, leading to high rates of missed diagnoses, while traditional diagnostic methods (such as colonoscopy and conventional CT scans) suffer from high invasiveness or insufficient contrast. Single treatment approaches (surgery, radiotherapy, or chemotherapy) are prone to side effects and have limited efficacy, making it difficult to balance tumor targeting with therapeutic synergy. Nanozyme-mediated “diagnosis-treatment integration” strategies offer novel solutions to this dilemma, but require overcoming bottlenecks including poor stability, separation of imaging and therapeutic functions, and lack of multi-mechanism coordination. Mei *et al*. 67 synthesized spherical CeO_2_ via hydrothermal synthesis, reduced HAuCl_4_ with sodium citrate to grow Au nanoparticles *in situ* on the CeO_2_ surface, dispersed CeO_2_@Au in IP6 solution, and formed a negatively charged organic shell layer through phosphate group chelation with metal ions. This resulted in a metal nanoenzyme CeO_2_@Au@IP6 (CeAIP). CeAIP enables high-contrast CT localization and real-time photothermal imaging via Au NPs, providing dual imaging support for precise diagnosis and image-guided multimodal therapy of colorectal cancer.

#### 2.6.4. For bimodal bioimaging of optical imaging and CT

Biomedical imaging demands deep tissue localization accuracy and cellular-level sensitivity, but CT (relying on high-atomic-number elements for high resolution) lacks cellular sensitivity, while traditional optical imaging (e.g., fluorescence) suffers from tissue autofluorescence, photobleaching, short lifetimes, and surface quenching. Quantum dots have stable luminescence but contain toxic metals (e.g., cadmium), and inorganic luminescent materials (e.g., rare-earth-doped aluminates) lose efficiency due to surface defects, limiting dual-modality imaging. An ideal platform also requires biocompatibility and dispersion stability to avoid *in vivo* aggregation. Calatayud *et al*. 68 fabricated a core-shell CeO_2_@Eu,Dy:SrAlO structure by *in situ* growing a ~10 nm CeO_2_ shell on Eu^2+^/Dy^3+^ co-doped strontium aluminate (SrAlO) luminescent nanosheets. The CeO_2_ shell—rich in high-atomic-number Ce (Z = 58)—exhibits strong X-ray attenuation (validated by X-ray diffraction) for CT imaging, enabling deep tissue localization when combined with the luminescent core. For optical imaging, the CeO_2_ shell suppresses surface quenching; in living CHO cells, the fluorescence lifetime second component (τ_2_) of CeO_2_@Eu,Dy:SrAlO prolonged from 2.34 ns (bare core) to 2.50 ns (core-shell), enhancing FLIM signal stability. Post-encapsulation, the material retains long-lived phosphorescence (>100 ns) for high signal-to-noise ratio lifetime imaging with low background interference. Under 800 nm two-photon excitation, the coated nanosheets generate detectable lifetime signals, suitable for deep tissue imaging and cell tracking. CeO_2_ in CeO_2_@Eu,Dy:SrAlO serves as a CT contrast agent for deep imaging and enhances the luminescent core's lifetime and stability via shell engineering, providing an integrated platform for nanoscale biological imaging.

#### 2.6.5. For display devices and LEDs, fluorescent lamps, etc

Stable, efficient multiband emission is core for luminescent materials in displays (e.g., LEDs, fluorescent lamps) and bioimaging. With a cubic fluorite structure, excellent chemical stability and ionic conductivity, CeO_2_ serves as an ideal luminescent host. However, its 4f orbitals are electron-deficient, which leads to weak intrinsic luminescence and necessitates rare-earth ion doping to introduce active centers. Er^3+^ ions, with unique 4f energy levels, enable visible/near-infrared (NIR) luminescence via energy transitions and can be excited by low-cost commercial laser diodes, making them preferred dopants. Chandrakar *et al*. 69 enhanced CeO_2_ NPs' luminescence by Er^3+^ doping. Under 251 nm UV excitation, the NPs emitted intense blue light (main peak 413 nm) with additional peaks at 470 and 594 nm (visible spectrum), suitable for displays (LEDs, FL, CFL) and fluorescence imaging. Under 980 nm infrared excitation, they exhibited NIR emission (848, 870, 980 nm), applicable for NIR fluorescence imaging or tissue penetration imaging. Though no specific bioimaging experiments were conducted, their visible/NIR emission properties highlight significant potential for optical imaging, with proven practical value in displays and future extensibility to bioimaging.

#### 2.6.6. For near-infrared fluorescence imaging-guided tumor therapy

To achieve integrated fluorescence imaging guidance and synergistic SDT/CDT combination, while enhancing treatment precision and enabling real-time drug distribution tracking, Xu *et al*. 70 constructed a multifunctional nanoplatform (CC@PP) by coating CeO_2_ and Ce6 with PEG-PLGA (polyethylene glycol-polylactic-polyhydroxyacetic acid copolymer) via the nanoprecipitation method. Functionalized CeO_2_ NPs, co-loaded with Ce6, acquired near-infrared fluorescence imaging capability, demonstrating excellent performance in tumor-targeted imaging, drug distribution monitoring, and treatment guidance. The fluorescence signal from Ce6 enabled dynamic monitoring of CC@PP distribution and metabolic processes *in vivo*. *In vivo* experiments revealed that after tail vein injection of CC@PP in mice, fluorescence signals progressively intensified in tumor regions at 6h, 12h, and 24h post-injection, peaking at 24h. This demonstrates effective tumor tissue accumulation of CC@PP and provides temporal guidance for subsequent ultrasound therapy. The imaging application of CC@PP not only enhances treatment visualization and precision but also lays the foundation for future multimodal imaging (e.g., fluorescence + ultrasound + photoacoustic) and expands its application scope.

## 3. Biodistribution and delivery strategies of functionalized CeO_2_ NPs

The therapeutic efficacy and biosafety of functionalized CeO_2_ NPs are highly dependent on their *in vivo* biodistribution, clearance rate, and administration route, which represent a critical conceptual gap in the current understanding of CeO_2_-based nanomedicines. A clear distinction between systemic administration and local delivery is essential to optimize targeted accumulation, minimize off-target effects, and promote clinical translation 71.

### 3.1. Systemic administration

Systemic infusion (e.g., intravenous injection) is the most common route for nanomedicine delivery, but it faces inherent bottlenecks for CeO_2_ NPs. Unmodified or poorly functionalized CeO_2_ NPs tend to rapidly accumulate in the reticuloendothelial system, particularly the liver and spleen, due to non-specific phagocytosis 72. Surface modification strategies such as PEGylation can prolong blood circulation time by reducing opsonization and immune clearance, but they rarely guarantee efficient delivery to pathological target tissues (e.g., solid tumors, inflamed intestinal mucosa, or injured neural tissues). This mismatched biodistribution leads to low local drug concentration, compromised therapeutic outcomes, and potential systemic toxicity caused by reticuloendothelial system accumulation 73.

### 3.2. Local delivery

Local delivery strategies outperform systemic administration by achieving high local concentration, reduced systemic exposure, improved therapeutic index, and lower dosage requirements, making them highly suitable for cutaneous 74, ocular 75, and local inflammatory lesions 76. Several representative local delivery platforms have demonstrated remarkable translational value for functionalized CeO_2_ NPs:

Gastrointestinal (GI) retention oral delivery systems are designed to address the shortcomings of oral nanomedicines for intestinal diseases (e.g., IBD), which are limited by rapid clearance and low mucosal retention. Specifically, the SHIELD system is fabricated by incorporating C18 ligand-functionalized CeO_2_ NPs into acid-treated sucralfate, forming a cohesive coacervate that uniformly coats the intestinal mucosa. As an oral nanoparticle aggregate, the SHIELD system forms a continuous protective coating over the full gastrointestinal tract, effectively covering widely distributed areas including inflamed regions. In colitis and radiation enteritis models, this system significantly enhances local therapeutic effects while effectively restricting systemic exposure, addressing the core contradiction between oral delivery and systemic toxicity 77.

Microneedle-based local delivery platforms Microneedle patches enable direct, non-invasive delivery of CeO_2_ NPs to mucosal or cutaneous lesions. For example, delivery of metabolized elastin-complexed CeO_2_ NPs to oral ulcer sites via microneedles markedly improves drug retention on accessible mucosal surfaces, enhances local ROS scavenging and anti-inflammatory effects, and validates the superiority of local administration for superficial lesions 78.

Hydrogel-based carriers, such as GelMA 79 and collagen 80, have been developed for the local delivery of CeO_2_ NPs. Notably, certain hydrogel systems, including zwitterionic hydrogels 81, have been shown to provide a sustained-release microenvironment for CeO_2_ NPs. Such hydrogel systems have demonstrated therapeutic efficacy in various applications, including diabetic wounds 82, bone defects 83, and OA 84, where they facilitate synchronized tissue repair.

### 3.3. Translational significance of delivery optimization

Tailoring biodistribution and delivery routes is a prerequisite for the clinical translation of functionalized CeO_2_ NPs. Local delivery strategies minimize reticuloendothelial system accumulation and systemic side effects 71, while targeted modification (e.g., ligand conjugation, microenvironment responsiveness) further improves the precision of systemic delivery 85. Clarifying the relationship between delivery strategies, biodistribution, and therapeutic outcomes will bridge the gap between laboratory research and clinical application of CeO_2_-based nanomedicines.

## 4. Toxicity and safety concerns of functionalized cerium oxide nanoparticles

The toxicity and safety of functionalized CeO_2_ NPs fundamentally depend on the type of functionalization modification 86, exposure dose 87,88, and the biological system involved 89.

### 4.1. Cases of reduced toxicity

#### 4.1.1. Biocompatible carrier / Functional molecule loading modification: Reducing CeO_2_ exposure to mitigate toxicity

CeO_2_ is a highly efficient ROS scavenger, yet its positive charge, poor gastrointestinal stability and easy intestinal absorption cause systemic exposure risks when used alone. Montmorillonite (MMT), an intestinal mucosal protector with negative charge, strong gastrointestinal stability and non-absorbability by the human body, can target positively charged inflammatory sites via electrostatic interactions but has no anti-inflammatory activity. To integrate their merits and overcome their drawbacks for an IBD therapy with oral stability, inflammation targeting, and low toxicity plus high efficacy, Zhao *et al*. 22 prepared CeO_2_@MMT nanozymes by in-situ growth of CeO_2_ NPs on MMT sheets. MMT's negative charge modified CeO_2_'s surface, which not only enhanced CeO_2_'s stability and dispersion but also reduced its gastrointestinal systemic absorption, thus alleviating potential nanotoxicity. The nanozymes' structure and composition were characterized by TEM, XRD and XPS. Regulating the CeO_2_/MMT ratio optimized the nanozyme composition, improved its gastric acid stability and enhanced oral performance. CeO_2_@MMT (1:9) exhibited favorable therapeutic effects and low toxicity in both *in vitro* and *in vivo* models. This functional design offers a vital reference for developing novel nanomedicines, proving that material compounding and surface modification can effectively reduce nanomaterial toxicity and boost their biomedical application potential.

Bone marrow mesenchymal stem cell-derived exosomes (BMSC-exos) serve as natural delivery vehicles, exhibiting not only excellent biocompatibility and immune evasion capabilities but also being rich in polyunsaturated fatty acids (PUFAs). They can assist in blocking lipid peroxidation chain reactions and enhance tissue penetration and targeted accumulation through their biomembrane properties. To integrate the antioxidant activity of CeO_2_ with the delivery advantages of BMSC-exos, addressing the therapeutic limitations of single-component approaches. Meng *et al*. 90 created a composite nanostructure, BMSC-exos@CeO_2_, by loading CeO_2_ NPs into bone marrow mesenchymal stem cell-derived exosomes (BMSC-exos), achieving functional modification of CeO_2_ NPs via natural exosomes. Compared to direct use of CeO_2_ NPs, BMSC-exos@CeO_2_ exhibited significantly reduced cytotoxicity. *In vitro* experiments using the mouse hepatocellular carcinoma cell line Hepa 1-6 demonstrated that BMSC-exos@CeO_2_ markedly alleviated Deoxynivalenol (DON)-induced cellular damage, oxidative stress, and inflammatory responses, outperforming either BMSC-exos or CeO_2_ alone. *In vivo* experiments in C57BL/6J mice demonstrated that BMSC-exos@CeO_2_ stably persisted in the liver for 72 hours with gradual metabolism, reducing accumulation risks. Compared to CeO_2_ NPs, it exhibited higher and more stable concentrations in the liver, indicating superior targeting and retention properties. BMSC-exos@CeO_2_ significantly reduced alanine aminotransferase/aspartate aminotransferase (ALT/AST) levels, protected liver function, suppressed inflammatory responses, modulated AMPK and JAK1/STAT3 signaling pathways, improved lipid metabolism and energy homeostasis, without inducing additional toxicity. Concurrently, the negative surface charge (-21.57 mV) of BMSC-exos@CeO_2_ reduces non-specific adsorption, prolongs circulation time, and ensures excellent dispersion with uniform particle size after exosome encapsulation, enhancing stability and tissue permeability. The exosome membrane structure possesses immune evasion capabilities, lowering the risk of immune clearance. BMSC-exos@CeO_2_ maintains potent antioxidant and anti-inflammatory activity while significantly reducing cytotoxicity, immune clearance risk, and *in vivo* accumulation risk, achieving a “safer and more effective” nanomedicine delivery system. However, future studies should conduct long-term toxicity research and preclinical safety assessments to ensure safety at higher doses or during prolonged use.

#### 4.1.2. Surface property modification: Mitigating the bioaccumulation toxicity of CeO_2_

Adding natural organic matter such as HA to the exposure medium of CeO_2_ NPs significantly reduces their toxicity. When the HA concentration is sufficiently high relative to CeO_2_ NPs, the bioaccumulation of cerium decreases 91. This is attributed to surface property modification: HA adsorbs onto positively charged CeO_2_ NPs, reversing their surface charge from positive to negative and promoting nanoparticle aggregation. These changes mitigate the electrostatic attraction between the nanoparticles and negatively charged biological membranes (e.g., the cuticle of Caenorhabditis elegans), thereby reducing cellular uptake and subsequent bioaccumulation and toxicity.

### 4.2. Cases of increased toxicity

#### 4.2.1. Surface positive charge modification

Researchers synthesized CeO_2_ NPs with positively charged, negatively charged, and neutral coatings, finding that positively charged CeO_2_ NPs exhibited significantly higher toxicity and greater bioaccumulation in Caenorhabditis elegans 91. Another study exposed C. elegans to cerium oxide nanomaterials coated with cationic polymer (DEAE), anionic polymer (CM), and nonionic polymer (DEX). The mortality and reproductive toxicity of DEAE-CeO_2_ were approximately one order of magnitude higher than those of CM-CeO_2_ or DEX-CeO_2_^92^.

#### 4.2.2. Specific intracellular localization

CeO_2_ NPs with positively charged or neutral surfaces can enter most studied cell lines. When localized within the lysosomes of cancer cells, they exhibit significant cytotoxicity, whereas toxicity is minimal when localized in the cytoplasm or when the nanoparticles fail to enter the cells 93.

## 5. Challenges in the functionalization of CeO_2_ NPs

To address the various challenges facing the biomedical translation of functionalized CeO_2_ NPs, it is necessary to gain a deep understanding of how particle size, morphology, surface chemistry, protein crown formation, *in vivo* distribution, and the conflicting effects of functionalization collectively determine the safety and efficacy of CeO_2_ NPs.

### 5.1. Size, morphology, and facet effects

Particle size is a dominant challenge for functionalized CeO_2_ NPs. Clearance depends critically on diameter: particles < 6 nm undergo rapid renal excretion, while larger ones are retained in the liver and spleen via hepatobiliary clearance, raising risks of chronic accumulation. Although a 2-10 nm window with optimal coating appears relatively safe in short-term studies, small particles may cross biological barriers (e.g., the blood-brain barrier) unpredictably, whereas larger ones tend to aggregate and trigger persistent macrophage activation. Moreover, size and surface functionalization interact non-linearly: a suitable coating cannot compensate for an inappropriate core diameter. Thus, balancing rapid clearance with minimal off-target retention and stable functionalization remains a major hurdle, requiring case-by-case optimization rather than a universal solution 71.

Beyond size, the morphology and exposed crystal facets of CeO_2_ NPs profoundly influence their enzyme-mimicking activities. CeO_2_ nanocubes with exposed {100} facets exhibit higher POD-like but lower SOD-like activity than nanorods with exposed {110} facets, despite comparable Ce^3+^/Ce^4+^ ratios and oxygen vacancy contents 30. This indicates that the spatial arrangement of surface Ce^3+^ sites, rather than their total concentration, dictates catalytic preference. Consequently, a one-size-fits-all surface modification strategy cannot be assumed to yield predictable biological effects across different CeO_2_ morphologies.Furthermore, Pütz *et al*. showed that for mesoporous CeO_2-x_, the Ce^3+^/Ce^4+^ ratio scales inversely with ζ-potential, allowing selective tailoring of haloperoxidase- and POD-like activities 94.

### 5.2. Surface chemistry, protein corona, and cellular interactions

The surface chemistry of CeO_2_ NPs determines not only their colloidal stability but also their biological identity upon contact with physiological fluids, a phenomenon mediated by the protein corona. Mazzolini *et al*. demonstrated that CeO_2_ NPs in serum-containing media acquires a protein corona enriched in transferrin, which mediates cellular entry via clathrin-mediated endocytosis. Under these conditions, CeO_2_ NPs does not affect cell growth, viability, or metabolism even at high concentrations. Conversely, in serum-free conditions, the same nanoparticles induce plasma membrane disruption and cause significant changes in cellular metabolism 95. This finding carries profound implications for the interpretation of *in vitro* toxicity studies of functionalized CeO_2_ NPs: results obtained in serum-free media may not accurately reflect the biological response *in vivo*, where protein corona formation is inevitable. Functionalization strategies must therefore be evaluated in physiologically relevant media to account for corona-mediated alterations in cellular uptake and toxicity.

The surface charge of CeO_2_ NPs also dictates their subcellular localization and consequent cytotoxicity. Asati *et al*. found that positively charged CeO_2_ NPs are predominantly localized within lysosomes of cancer cells, where they exhibit significant cytotoxicity, whereas negatively charged or neutral NPs show minimal toxicity when localized in the cytoplasm or when failing to enter cells 93. This charge-dependent intracellular trafficking pathway suggests that the same functionalized CeO_2_ NPs may exhibit drastically different toxicological profiles depending on the surface charge imparted by the modification strategy. Such an outcome is difficult to predict a priori without detailed mechanistic studies.

### 5.3. Biodistribution, metabolism, and clearance

The *in vivo* behavior of CeO_2_ NPs is characterized by substantial inter-individual and inter-strain variability, largely attributable to differences in immune status and reticuloendothelial system activity 96. Yokel *et al*. demonstrated that intravenously administered 30 nm CeO_2_ NPs are primarily retained in organs of the mononuclear phagocyte system, including the liver, spleen, and bone marrow. Over 90 days, there was little decrease in tissue cerium content, and this retention was accompanied by granuloma formation in the liver 72. Ernst *et al*. further reported that 3 nm CeO_2_ NPs accumulate in the spleen and liver, with exponential decay such that approximately 50 % of the nanoparticles are excreted within 100 days, primarily via urinary (cerium ions) and hepatobiliary (nanoparticles) routes 97. The long-term persistence of CeO_2_ NPs in RES organs, lasting months after a single administration, poses a significant challenge for functionalization strategies aimed at reducing systemic accumulation. The rate and pathway of clearance are further modulated by particle size, coating, and surface chemistry, but systematic structure-clearance relationships remain to be established.

### 5.4. Paradoxical effects of functionalization: from protection to new sources of toxicity

Functionalization is widely employed to improve the dispersion, stability, and targeting of CeO_2_ NPs, yet emerging evidence indicates that such modifications can paradoxically introduce new toxicity mechanisms or exacerbate adverse effects relative to the unmodified nanoparticles. Fisichella *et al*. conducted a combined physicochemical and whole-genome expression analysis of CeO_2_ NPs before and after simulated environmental or gastrointestinal degradation 86. They found that citrate-coated CeO_2_ NPs, upon degradation by light or gastric acid, partially shed their protective surface coating, exposing a “bare core.” This exposed core induced significantly enhanced gene expression disruption and mitochondrial dysfunction, exhibiting toxicity patterns closer to those of uncoated CeO_2_ rather than the intact functionalized particles. This study suggests that the degradation products of functionalized CeO_2_ may exhibit greater toxicity than the parent nanoparticles. Notably, this observation is especially relevant for oral formulations, which inevitably come into contact with the acidic environment of the stomach.

The role of surface functionalization in altering the *in vivo* fate of CeO_2_ NPs has been further explored by Rahman *et al*., who demonstrated that biomineralization on a bovine serum albumin substrate produces CeO_2_ NPs that passively target inflammatory sites via albumin's properties, sparing healthy tissues. While this functionalization strategy confers targeting advantages, it simultaneously introduces new variables, including protein conformation, surface charge, and aggregation state. These variables are difficult to control across batches and may unpredictably influence immunogenicity and clearance 46.

Surface charge-mediated toxicity represents another paradoxical outcome of functionalization. Arndt *et al*. exposed C. elegans to CeO_2_ nanomaterials coated with cationic polymer (DEAE), anionic polymer (CM), and nonionic polymer (DEX) 92. The EC_30_ for reproductive toxicity of DEAE-CeO_2_ was approximately two orders of magnitude lower than that of CM-CeO_2_ or DEX-CeO_2_. These findings collectively indicate that the biological outcome of functionalized CeO_2_ NPs depends on a delicate balance of surface properties, where modifications intended to enhance biocompatibility may inadvertently increase toxicity if the surface charge is not carefully optimized.

The formation of a protein corona on functionalized CeO_2_ NPs further complicates this picture. While protein corona formation generally attenuates direct membrane damage by providing a biological interface, corona composition varies with surface functionalization. Certain proteins become enriched within the corona, which can redirect cellular uptake pathways and alter intracellular trafficking 95. For example, transferrin in the corona facilitates clathrin-mediated endocytosis, which may bypass certain degradation pathways and prolong nanoparticle retention. Functionalization strategies that unintentionally alter corona composition thus risk producing unanticipated long-term accumulation and chronic toxicity profiles.

### 5.5. Challenges in large-scale preparation and cost control

The production of high-quality, reproducible functionalized CeO_2_ NPs still relies on complex synthesis routes such as sol-gel and hydrothermal methods, which suffer from significant batch-to-batch variability, high costs, and substantial environmental pressures. Ahamed *et al*. highlighted that persistent challenges in CeO_2_-based nanomaterials include stabilizing oxygen vacancies under realistic conditions and achieving scalable application in environmental and biomedical technologies 23.

## 6. Conclusion

Functionalized CeO_2_ NPs hold broad application prospects in the biomedical field, demonstrating outstanding potential in areas such as anticancer, anti-infection, anti-inflammatory, antioxidant activity, wound healing, and neuroprotection.

Despite the tremendous potential of CeO_2_ NPs in biological applications, further research is still needed on their long-term effects *in vivo*, toxicity, and optimal delivery systems. Future approaches may involve reducing toxicity through improved synthesis methods and surface modifications, enabling the customization of CeO_2_ NPs with minimal or no toxicity to biological systems. Biocompatible polymer coatings and/or targeted CeO_2_ NPs could enhance therapeutic activity by minimizing side effects and improving pharmacokinetics. Furthermore, delving into its mechanism of action remains a key focus for future research.

## Figures and Tables

**Table 1:** Typical functionalization modification strategies for cerium oxide nanoparticles.

Strategies	Type of modifier	Core modifier	Core role	Application scenarios	Reference
Polymeric organic ligands	Natural polymers	Dextran	Increase intracellular ROS levels to induce apoptosis in head and neck cancer cells	*In vitro* Treatment Research for Head and Neck Cancer	(34)
Bioorganic ligands	Amino acid	Histidine, glycine	Enhance antiviral activity by targeting and disrupting viral entry	Prevention and control of enveloped virus infections *in vitro*	(41)
	Nucleic acid	miR146a	Targeted inhibition of inflammatory signaling pathways, efficient clearance of ROS	Treatment of chronic IBD	(44)
	ECM-derived structural proteins	mEl	Stabilizes dispersed CeO_2_, enhancing ROS scavenging and promoting angiogenesis	Microneedle Therapy for Oral Ulcers	(78)
Small-molecule organic ligands	Organic acids	PA	Chelating excess intracellular Ca^2+^ and inhibiting P-gp expression to reverse tumor resistance	Chemotherapy Sensitization and Toxicity Mitigation in Doxorubicin-Resistant Hepatocellular Carcinoma	(39)
Inorganic nanocomposites	2D MoS_2_ nanosheet	PEG-MoS_2_ nanosheets	Highly efficient photothermal antibacterial capability, enhanced antioxidant activity	Chronic wound treatment, particularly diabetic ulcer wounds	(28)
	2D graphene nanoplatelet	GNPs	Synergistically enhances antimicrobial activity	Prevention and control of multidrug-resistant bacterial infections	(40)

**Table 2:** Synthesis methods for functionalized modification of cerium oxide nanoparticles in different application scenarios.

Synthesis method	Models	Main results	Applications	Reference
Precipitation Synthesis of Dex-CeO_2_ NPs	Cells: head and neck cancer cell lines A253, FaDu, SCC-25	SD2 exhibits superior anticancer activity and stability, exerting its effects through the apoptosis pathway	Treatment of Head and Neck Cancer	(34)
Solvothermal synthesis of G/CeO_2_ nanotiles loaded with 12% and 25% GNPs	Bacteria:P. aeruginosa, S. aureus	G/CeO_2_-II (25% GNPs) exhibited the most optimal antibacterial properties, achieving inhibition rates of 82.67% and 89.48% against the two bacteria, outperforming pure CeO_2_	As a novel nano-antibiotic for controlling multidrug-resistant bacteria	(40)
Conjugation of miR146a to CeO_2_ NPs via CDI chemistry	Animals:Establishing a Chronic Colitis Model Induced by T Cell Transplantation in Rag2^-^/^-^ Mice	CNP-miR146a is orally stable and significantly alleviates colitis symptoms	Treatment of IBD	(44)
Hydrothermal synthesis of CeO_2_ nanospheres, reduced with hydrazine hydrate to load Cu and Pt, constructing PtCuO_x_/CeO_2-x_	Cells: SD rat primary chondrocytes (IL-1β-induced inflammation) Animals: SD rat ACLT osteoarthritis model	PtCuO_x_/CeO_2-x_ exhibits high SOD/CAT activity, scavenging ROS/RNS, protecting mitochondria, and inhibiting the ROS/Rac-1/NF-κB pathway	Treatment of OA	(13)
Grow cerium oxide nanoclusters on BSA via biomineralization, then couple them with ICG.	Cells: J774 Macrophages Animals: Uterus-Specific Stat3 Knockout Mice (Pgrre/+Stat3f/f; Stat3d/d); Endometriosis Mouse Model	CeO_2_ can target ectopic lesions in mouse endometriosis, scavenge ROS, modulate immunity, inhibit pSTAT3, reduce lesions without affecting pregnancy, and enable non-invasive imaging when conjugated with ICG	Targeted Therapy for Endometriosis	(46)
Disperse CeO_2_ in water, add DA to adjust the pH to 9.0, and undergo oxidative polymerization to form Ce@P nanoenzymes coated with PDA	Cells: RAW264.7 macrophages (LPS-induced inflammation) Animals: SD rats administered LPS via tracheal instillation to establish an ALI model	Ce@P+NIR can efficiently scavenge ROS, reduce inflammatory factors, induce M2 polarization, and alleviate lung injury	Targeted Therapy for ALI	(47)
A one-step hydrothermal process enables α-CD to encapsulate cerium oxide via O-Ce bond formation, yielding CCNP. Subsequently, MOR is loaded through intermolecular interactions between α-CD and MOR	Cells: RAW264.7 macrophages (LPS-induced inflammation); Animals: C57BL/6 mice with laser-induced CNV model	M@ CCNP can eliminate ROS, inhibit M1 polarization, activate NRF2, reduce CNV area by 84.4%, and decrease leakage by 68.4%, demonstrating superior efficacy in suppressing leakage compared to anti-VEGF drugs	Treatment of AMD	(48)
Chemically synthesized CeO_2_ was ultrasonically mixed with HA solution, followed by ethanol precipitation and centrifugation to obtain HA-CeO_2_	Cells:HCECs; Animals:C57BL/6 mice	HA-CeO_2_ effectively scavenges ROS, reduces IL-1β/MMP9, increases tear volume and tear film stability, and repairs the cornea	Treatment of DED	(49)
Aminated CeO_2_ was first prepared via silylation with APTES, followed by chemical coupling with ALG using EDC/NHS to construct Ce-ALG nanoparticles	Cells: Rabbit corneal epithelial cells (SIRC, LPS/H_2_O_2_-induced inflammation/oxidative stress); Animals: New Zealand white rabbits used to establish a CA model	Ce-ALG enhances adhesion and cellular uptake, enabling controlled release of β-1,3-glucan. It achieves 99% repair of abrasions, outperforming ketorolac and unmodified Ce	Treatment of CA	(50)
Solvothermal synthesis of CeO_2_ NPs fused with Pltm via ultrasonication and embedded in GelMa hydrogels yields Pltm@CeO_2_ NPs/Gel	Cells: HaCaT keratinocytes (H_2_O_2_-induced oxidative stress), RAW264.7 macrophages (LPS-induced inflammation); Animals: GK rats used to establish a diabetic wound model	Pltm@CeO_2_ NPs/Gel can scavenge ROS, suppress inflammation, promote angiogenesis and collagen alignment, significantly accelerating wound healing in diabetic rats	Diabetic Wound Care	(52)
CeO_2_ nanoparticles were mixed with a GelMA solution containing bone marrow-derived mesenchymal stem cells (BMSCs), and UV-cured to produce a GelMA-CeO_2_-BMSC composite hydrogel	Cells: BMSCs, RAW264.7 macrophages; Animals: SD rats used to construct a 5mm cranial critical defect model	GelMA-CeO_2_-BMSC promotes osteogenic differentiation of BMSCs, suppresses M1 polarization while promoting M2 polarization, and significantly repairs rat cranial defects	Repair and Regeneration of Bone Defects	(54)
PAA is modified on the surface of CeO_2_ to synthesize CeO_2_@PAA, which is then conjugated with biotinylated LXW7 via EDC reaction to construct CeO_2_@PAA-LXW7	Cells: Mouse BV2 microglia, established as an inflammatory model by LPS induction	CeO_2_@PAA-LXW7 can scavenge ROS/NO, reduce TNF-α/IL-1β, and inhibit the phosphorylation of FAK and STAT3, exhibiting superior anti-inflammatory effects compared to CeO_2_@PAA or LXW7 used alone	Interventions for Neuroinflammation Associated with Central Nervous System Injury	(56)
After synthesizing CeO_2_ nanoclusters, the acidified ZnTPyP is self-assembled on their surface via hydroxyl interaction, and PLL is modified to obtain CZPN	Cells: Rat pheochromocytoma PC12 cells; Animals: ICR mice	CZPN can optically modulate neuronal activity, ensuring cellular safety, and induces TRP channel-mediated pain behavior in ICR mice	Treatment of Peripheral Nerve Disorders	(64)

**Table 3:** Synthesis methods, mechanism improvements, and biomedical applications of typical CeO_2_-based heterostructure nanoenzymes.

Types of heterostructures	Synthesis Method	Key Mechanism	Representative biomedical applications	Reference
CeO_2_/Mn_3_O_4_ heterostructure	Epitaxial growth method	Lattice strain-induced formation of oxygen vacancies; Enhanced ROS scavenging capacity; Improved radiation protection efficacy	Radiation Protection	(27)
CeO_2_/MoS_2_ heterostructure	Solvothermal method + PEG modification	MoS_2_ provides photothermal antibacterial activity; CeO_2_ scavenges ROS for antioxidant and wound healing promotion	Healing of chronic wounds (such as diabetic ulcers)	(28)
CeO_2_/Au@Pt heterostructure	Seed-growth method + reduction deposition	CeO_2_ nanozymes with POD/CAT-like activities; Au@Pt provides photothermal effect; synergistic photothermal-catalytic tumor therapy	Photothermal-catalytic synergistic therapy for tumors	(29)
PtCuO_x_/CeO_2-x_ heterostructure	Hydrothermal synthesis + reduction doping (Cu, Pt)	Cu doping increases the Ce^3+^/Ce^4+^ ratio and creates oxygen vacancies; Pt stabilizes Cu⁺ and enhances SOD/CAT-like activity; NIR response enhances catalytic efficiency	Treatment of OA	(13)

**Table 4:** Effects of functionalization strategies on enzyme-mimetic activities of CeO_2_ NPs.

Functionalization strategies	Typical Materials	SOD-like activity	CAT-like activity	POD-like activity	Key mechanisms	Key Use Cases	References
Element doping	PtCuO_x_/CeO_2-x_	Significantly enhanced	Significantly enhanced	—	Co-modification with a Cu-Pt bimetallic system increases the Ce^3+^/Ce^4+^ ratio, raises the oxygen vacancy concentration, and lowers the oxygen vacancy formation energy	Treatment of OA	(13)
Surface Modification	CeO_2_/Au@Pt-PEG	—	Significantly enhanced	Significantly enhanced	A stable core-shell Au@Pt structure effectively supports ultra-small CeO_2_ particles, addressing the issue of nanoenzyme aggregation; PEG modification enhances the material's water dispersibility, biocompatibility, and circulation time *in vivo*; the Au@Pt component provides highly efficient near-infrared photothermal conversion capabilities, enabling photothermal therapy	Photothermal-catalytic synergistic therapy for tumors	(29)
	HA-CeO_2_	—	Significantly enhanced	—	HA modification improves the dispersion stability and biocompatibility of CeO_2_, enhances its ability to adhere to and remain on the ocular surface, increases tear film thickness and stability, and exerts synergistic antioxidant and anti-inflammatory effects	Treatment of DED	(49)
	HA-5-HT@CeO_2_	—	Enhanced	—	HA targets CD44^+^ macrophages; 5-HT acts as a substrate for MPO in inflamed colon, enabling targeted accumulation; CeO_2_ scavenges ROS (H_2_O_2_, •OH) and reduces pro-inflammatory cytokines (TNF-α, IL-6, IL-1β) while upregulating anti-inflammatory IL-10; repairs intestinal epithelial barrier (upregulates ZO-1, occludin, E-cadherin)	Treatment of UC	(43)
	M@CCNP	Significantly enhanced	Significantly enhanced	—	α-CD binds to the CeO_2_ surface via O-Ce bonds, enhancing the dispersibility, stability, and biocompatibility of the nanoparticles; MOR activates the NRF2 pathway and inhibits NF-κB, downregulating pro-inflammatory factors, suppressing M1 polarization of macrophages, and blocking the inflammatory cascade in AMD	Treatment of AMD	(48)
	Ce@P	Significantly enhanced	Significantly enhanced	—	PDA coating enhances the water dispersibility, colloidal stability, and biocompatibility of CeO_2_; PDA endows the material with highly efficient NIR photothermal conversion capabilities, and the synergistic photothermal effect enhances the ROS scavenging efficiency of CeO_2_; Ce@P efficiently scavenges ·OH, ·O_2_^-^, and H_2_O_2_ upon NIR activation, thereby breaking the oxidative stress-inflammation vicious cycle	Treatment of ALI	(47)
Surface Facet Engineering	CeO_2_ nanocubes with {100} crystal faces exposed	lower	—	higher	{100} The {100} crystal plane has high surface energy and a high degree of reconstruction, resulting in stronger POD-like catalytic activity	Catalytic Applications	(30)
	CeO_2_ nanorods with {110} crystal faces exposed	higher	—	lower	{110} The crystal surface is more conducive to ·O_2_^-^ adsorption and protonation, resulting in superior SOD-like activity	Natural antioxidants	(30)

Note: “—” indicates that the literature does not explicitly report or focus on the activity of this type of enzyme.
